# Self‐Healing and Reprocessable Soft Robots Using 3D Digital Light Printing

**DOI:** 10.1002/advs.202516901

**Published:** 2025-11-03

**Authors:** Chenggang Yuan, Yuqing Qin, Miaomiao Liu, Wai Hin Lee, Mantas Drelingas, Sebastian Fieldhouse, Alan M. Wemyss, Poh Sheng Tay, David M. Haddleton, Chris Bowen, Chaoying Wan, Min Pan

**Affiliations:** ^1^ Department of Mechanical Engineering University of Bath Bath BA2 7AY UK; ^2^ International Institute for Nanocomposites Manufacturing (IINM) University of Warwick Coventry CV4 7AL UK; ^3^ Department of Chemistry University of Warwick Coventry CV4 7AL UK

**Keywords:** 3D digital light printing, additive manufacturing, reprocessable soft robots, self‐healing robots, soft robots

## Abstract

Soft robots manufactured from compliant materials are highly versatile and can interact safely with humans while performing complex tasks. However, their low modulus and high compliance make them vulnerable to mechanical damage. Here, we synthesise soft, self‐healing, and recyclable robots featuring complex air chambers using 3D digital light printing technology. The formulated monomers and cross‐linkers are polymerized layer‐by‐layer using photoinitiated free‐radical polymerization during the printing process to form soft objects on a moving metal substrate. Dynamic chemistry is introduced into the polymer by designing cross‐linker structures, whereby vinylogous urethanes‐bearing cross‐linkers of different chain lengths are studied to allow the cross‐linked elastomer networks to be thermally triggerable for self‐healing and reprocessing. The resultant elastomer exhibits a tensile strength of 3.51 ± 0.1 MPa and an elongation at break of 454 ± 56% with optimized formulations and printing parameters. The printed soft grippers and crawlers are investigated for their static and dynamic performance after being punctured, cut in half, and left to self‐heal at room temperature for 24 h. They exhibit excellent self‐healing capabilities with efficiencies of 94.5% and 87.5%, respectively. This new approach creates self‐healing, recyclable soft robots with complex geometries through additive manufacturing, enabling sustainable, resilient robots for challenging environments.

## Graphical abstract

This work advances 3D digital light printing of soft robots made from self‐healing, recyclable elastomers enabled by dynamic covalent bonds. With the introduction of room‐temperature healing, these robots recover their mechanical performance (94.5% static, 87.5% dynamic) after damage and maintain their functionality. The approach enables self‐healing, recyclable soft robots with complex geometries through additive manufacturing, thereby fostering sustainable and resilient robots for extreme environments.

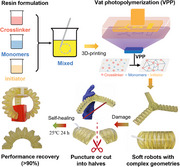

## 1 Introduction

Bio‐inspired soft robots fabricated from compliant materials with low elastic moduli in the range of natural organisms (10^4^–10^9 ^Pa) are needed in order to provide safe human interaction,^[^ ^1^, ^2^, ^3^ ^]^ high adaptability to harsh environments, and high levels of agility to achieve complex tasks. They can be designed to mimic the movements of living organisms, making them ideal for tasks that require dexterity, flexibility, and adaptability. Recently, new forms of soft robots have been developed for a diverse range of applications, including robotic surgery operations,^[^ ^4^, ^5^ ^]^ assistance and rehabilitation,^[^ ^6^, ^7^ ^]^ search and rescue,^[^ ^8^, ^9^ ^]^ exploration,^[^ ^10^, ^11^ ^]^ and industrial automation.^[^ ^12^, ^13^ ^]^ However, due to their soft nature and unique design, they are prone to various types of damage during operation. One of the major challenges with soft robots is their susceptibility to cuts, tears, and punctures from sharp objects. The low‐modulus materials employed to make soft robots often lack the durability of materials that are used in traditional rigid robots, which makes them more vulnerable to damage. In addition to physical damage, soft robots are prone to structural damage, such as interfacial debonding and rupture, as a result of overpressures, overloading, and repeated actuation. These forms of damage can occur when the robot is subjected to excessive force or cycles of operation, leading to the separation of different layers of the robot's structure. As a result, the robot may lose its shape and functionality, or even fail. Another type of damage that soft robots are susceptible to is electrical breakdown from a high voltage; for example, many soft robots use electrical signals to control their movements, and any disruption in the electrical circuit can cause the robot to malfunction. A high voltage can damage the internal components of the robots, leading to failure or even posing a safety risk. To increase lifetime, robustness, and sustainability, advanced soft robots with self‐healing capability are therefore desired to recover their performance after damage.

Soft self‐healing elastomers have attracted interest for soft robotic applications since they are capable of autonomously healing and recovering from mechanical and electrical damage, which can extend the lifetime of soft robots and devices.^[^ ^14^ ^]^ Intrinsic self‐healing materials have been developed based on non‐covalent dynamic bonds such as hydrogen bonding, metal‐ligand coordination, ionic interactions, host‐guest interactions, and covalent dynamic bonds such as Diels‐Alder reactions, imine bonds, disulfide bonds, and borate ester bonds.

Self‐healing materials have enabled the development of self‐healable robotic devices since the 2010s. Shepherd et al. blended polyaramid fibers into uncured silicone and created a pneumatic soft gripper that could seal a defect produced by a needle puncture.^[^ ^15^ ^]^ The gripper was able to rapidly recover its original shape after piercing and pressing the crack, allowing for sealing to proceed through material adhesion. Intrinsic self‐healable robotic hands, grippers, and actuators based on Diels‐Alder dynamic bonds were reported by Terryn et al.^[^ ^2^ ^]^ They were able to self‐heal large cuts with lengths up to 9.5 mm and perforations that were induced by an overpressure, after heating the material to a temperature of up to 80 °C for more than 24 h. A self‐healable heater was fabricated from the Diels–Alder reaction crosslinked elastomer filled with 20 wt.% carbon black to eliminate the need for an external heating source, which was embedded into a self‐healing finger actuator.^[^ ^16^ ^]^ The finger actuator was able to almost fully recover its performance at 90 °C in only 15 min after Joule heating from the heater. Self‐healing of these devices at room temperature (25 °C) was also implemented,^[^ ^17^ ^]^ however, a long healing time of 14 days to fully (97%) recover from cuts was required. Jiang et al. developed a pneumatically‐driven self‐healable crawling robot by embedding reversible dynamic imine bonds from triformylbenzene into a polydimethylsiloxane network.^[^ ^14^ ^]^ The synthesized elastomer was stretched to 2400% and recovered 89.6% of its original performance from cuts after self‐healing at room temperature for 24 h or 70 °C for 1 h. The robot could crawl at a speed of 150 mm min^−1^ and traverse slopes of up to 45 degrees; the robot deformation was unaffected after puncturing and self‐healing.

Wang et al. have developed a lightweight, robust, and light‐driven swimming robot with self‐healing capability at room temperature.^[^ ^18^ ^]^ This was achieved by creating a bioinspired gradient nanostructure between modified sulfonated graphene nanosheets in polyurethane. This structure forms high‐density hydrogen bonds, which enable self‐healing at room temperature, and the self‐healing capability was evaluated on a tensile test sample and for a fish‐shaped swimming robot. The sample was able to lift over 50 000 times its own weight and exhibited an 89% self‐healing efficiency in terms of ultimate tensile strength after cutting the sample in half and allowing it to self‐heal for 12 h at room temperature. The robot reached a maximum speed of 2.67 BL (body length)/s and could recover its swimming function after cutting and self‐healing under the same conditions. Chen et al. fabricated a soft self‐healable gripper from a self‐healable, recyclable, and degradable soft material based on hydrogen bonding and metal coordination interactions of a gelatin‐polyvinyl alcohol network.^[^ ^19^ ^]^ The material sample was able to recover 90.4% and 85.9% of its original fracture strain and stress, respectively, after being cut in half and self‐healing at 85 °C for 5 h. The fabricated gripper almost fully recovered its bending performance and grasping function after repeatedly cutting through the finger cross‐section and self‐healing. Guo et al. incorporated supramolecular halogen bonding into a liquid crystal elastomer (LCE) actuator to enable self‐healing and shape programmability.^[^ ^20^ ^]^ The actuator was stretched up to 580% and had a tensile strength of 2 MPa. When heated by light to 110 °C for 5 min, a self‐healed actuator reached 60% of its original performance. The actuators were deformed and programmed to a shape at 55 °C and then cooled to room temperature for 3 days to equilibrate. The actuator was reversibly switched between the deformed and original shapes through heating and cooling, which was enabled by the two‐way shape‐memory effect.^[^ ^21^ ^]^ Based on this approach, they developed a light‐driven walking robot with a speed of 0.05 body length (BL) /min and a rolling robot with a speed of 1.3 cm s^−1^ on a hot plate by stretching and twisting LCE strips, respectively. The rolling motion was retained after cutting and healing, which validated the self‐healing capability of LCE robots. A self‐healing 2D butterfly robot was developed by Zhu et al., which was able to transform into a 3D structure within 2 s using a magnetic field stimulus.^[^ ^22^ ^]^ The robot was based on magnetic Neodymium‐Iron‐Boron microparticles filled with polyamine composites, with dynamic covalently bonded interfacial bonding. After cutting in half, the composite exhibited a maximum strain of 260% and an ultimate tensile strength of 3.8 MPa and was able to heal within 10 min by adding a small amount of amine to the broken interface. The butterfly robot fully recovered its transformation capability after being cut into two parts and healed. A self‐healing spider‐shaped robot was manufactured from a magnetic elastomer with a hierarchical dynamic network, which consisted of disulfide bonds, hydrogen bonding, and metal‐ligand coordination interactions.^[^ ^23^ ^]^ The elastomer had a combination of good mechanical properties (maximum strain of 600% and ultimate tensile strength of 0.4 MPa) and fast healing (90% recovery rate at 20 °C for 5 min). The robot performed continued locomotion after being cyclically (every 10 s) cut into two halves and self‐healed for 5 s, showing excellent self‐healing speed and efficiency.

Dielectric Elastomer Actuators (DEAs) are a class of soft actuators that need to be stacked to achieve a large dielectric area to output high force, which increases the possibility of electrical breakdown events. Ellingford et al. created a self‐healing methyl thioglycolate‐grafted styrene−butadiene−styrene (MG‐SBS) dielectric elastomer that achieved a high tensile strength of 3.13 MPa, elongation at break of 569%, and relative dielectric permittivity of 11.4 at 1 kHz.^[^ ^24^ ^]^ A self‐healing crawling robot was fabricated by a melt‐extruded MG‐SBS elastomer extruded tube driven by a shape‐memory alloy spring, and was able to crawl at 1.57 BL/min.^[^ ^25^ ^]^ After being repeatedly cut into pieces, the robot recovered its locomotion capability by self‐healing at room temperature for 24 h. Cheng et al. developed an amphibious dielectrically actuated soft robot with self‐healable electrodes based on ionic conductors.^[^ ^26^ ^]^ The outer electrode layer of the robot could self‐heal and recover the robot's locomotion ability in 5 min, both in water and on land, after damage such as a small cut or being completely scratched off, without any external stimuli. Hydraulically‐amplified self‐healing electrostatic (HASEL) actuators, reported by Acome et al., are a new class of soft actuators that combine the versatility of fluid‐driven actuators and the high performance of dielectric actuators.^[^ ^27^ ^]^ HASEL actuators utilize an electric field to drive self‐healing dielectric fluid in a soft structure to generate a hydraulic pressure locally. The dielectric liquid self‐heals and recovers the gap instantly after an electrical breakdown. A soft gripper was developed from stacks of donut‐shaped actuators to demonstrate the grasping of fragile objects such as raspberries and eggs. To enhance the actuator performance, Tian et al. designed a bilayer‐structure HASEL actuator that achieves a large strain of 164% at 5 kV and a load‐bearing force of 620 mN at 6 kV for a single unit.^[^ ^28^ ^]^

Self‐healable soft actuators and robots are typically fabricated and manufactured using customized molding and casting. These methods are time‐consuming and labor‐intensive to design, fabricate, and assemble both the molds and soft robots, in particular for small‐number production and design iteration studies.^[^ ^29^ ^]^ These manufacturing methods also limit the available robot geometries and design space. For example, to fabricate hollow structures such as air chambers by molding and casting, the mold cores must be removed during demolding, which requires separating the soft robots into parts and assembling them using adhesives. Advanced and versatile manufacturing techniques are therefore necessary for the development of soft robotics. Recently, there have been promising commercialized additive manufacturing (3D‐printing) technologies, including selective laser sintering, stereolithography, direct ink writing, fused filament fabrication, and digital light processing^[^ ^30^ ^]^ that use flexible materials to manufacture soft robotic devices.^[^ ^31^, ^32^, ^33^, ^34^, ^35^, ^36^ ^]^ These technologies provide low‐cost and rapid prototype iteration, advanced design availability, a wide range of material selection, and high versatility for smart design and fabrication of soft robots. Despite the promising features of additive manufacturing, only a limited number of researchers investigate the additive manufacturing of self‐healable soft robots. Roles et al. implemented additive manufacturing on a Diels–Alder (DA) based self‐healable soft gripper using fused filament fabrication, also known as fused deposition modelling.^[^ ^30^ ^]^ The DA‐based material is extruded into 3D‐printing filaments, which are heated to a viscous liquid state and extruded by the nozzle to print the 3D objects. The 3D‐printed gripper can recover its shape from scratches and cuts after being healed at 90 °C for 30 min and placed at room temperature for 24 h. Zhang et al. fabricated various 3D‐printed self‐healable structures with a high resolution of up to 30 µm using digital light processing 3D‐printing technology.^[^ ^37^ ^]^ The 3D‐printed structure was able to return to its original shape by changing temperature due to the shape‐memory effect. They incorporated compatible semicrystalline linear polymer polycaprolactone (PCL) into a methacrylate‐based shape memory polymer system to realize the self‐healing function. The material exhibits a tensile strength and strain at break recovery of more than 90% with 20 wt.% of PCL in the composite after being healed for 20 min at 80 °C. A 3D‐printed thermal‐driven gripper was cut and healed at 80 °C for 5 min, and could recover its functions. Yu et al. fabricated a self‐healable soft actuator using a 3D‐printable photoelastomer with thiol and disulfide groups.^[^ ^38^ ^]^ The 3D‐printed photoelastomer sample exhibited over 90% self‐healing efficiency at 80 °C for 2 h. The vacuum‐driven soft actuator can lift 10 times its own weight at −30 kPa for 6 mm and retain its performance after being cut in half and healed at 60 °C for 2 h. Gomez et al. developed a self‐healable soft gripper by assembling 3D‐printed submodules using a vat photopolymerizable elastomer.^[^ ^39^ ^]^ The fabricated material achieved a high strain of 1500% with full strength recovery after being cut into two halves and healed at 90 °C for 24 h. The modular subcomponents of the gripper were 3D‐printed by digital light processing (DLP) and assembled by pressing and heating them to self‐heal. The gripper was able to grasp and lift a ball with a diameter of 12.7 cm and a weight of 142 g.

In summary, limited work has been undertaken on 3D printing of self‐healing soft robots and, to date, heating has often been employed to facilitate self‐healing and recovery of properties after damage. The potential to re‐use and re‐process the soft robot has also yet to be explored. In this work, we have therefore developed resilient, damage‐tolerant, and re‐processable soft robots that are able to self‐heal at room temperature and be formed into complex shapes by 3D printing, which are enabled by employing a dynamic bond exchange mechanism based on covalent vinylogous urethane. These soft robots can be directly 3D‐printed by commercialized 3D printers based on vat photopolymerization (VPP) and reused by cryogrinding and compression molding at 150 °C. The 3D‐printed elastomer samples exhibit a tensile strength of 3.51 ± 0.1 MPa and strain at a break of 454 ± 56% using optimized polymer formulation and 3D‐printing parameters. Self‐healing grippers and crawlers were prototyped and mechanically characterized, and their self‐healing capability was investigated in detail by subjecting them to severe punctures and cutting them in half. After self‐healing at room temperature for 24 h, the gripper and crawling robot exhibited excellent self‐healing efficiencies of 94.5% and 87.5%, respectively, considering both static and dynamic performances in detail, including frequency responses. This work therefore provides a new pathway for developing next‐generation resilient and damage‐tolerant soft robots with highly complex structures, customized geometry, efficient self‐healing capability, and ease of reuse for sustainable production.

## 2 Results

### 2.1 Vat Photopolymerization 3D Printed Self‐Healing Robots

**Figure 1:**
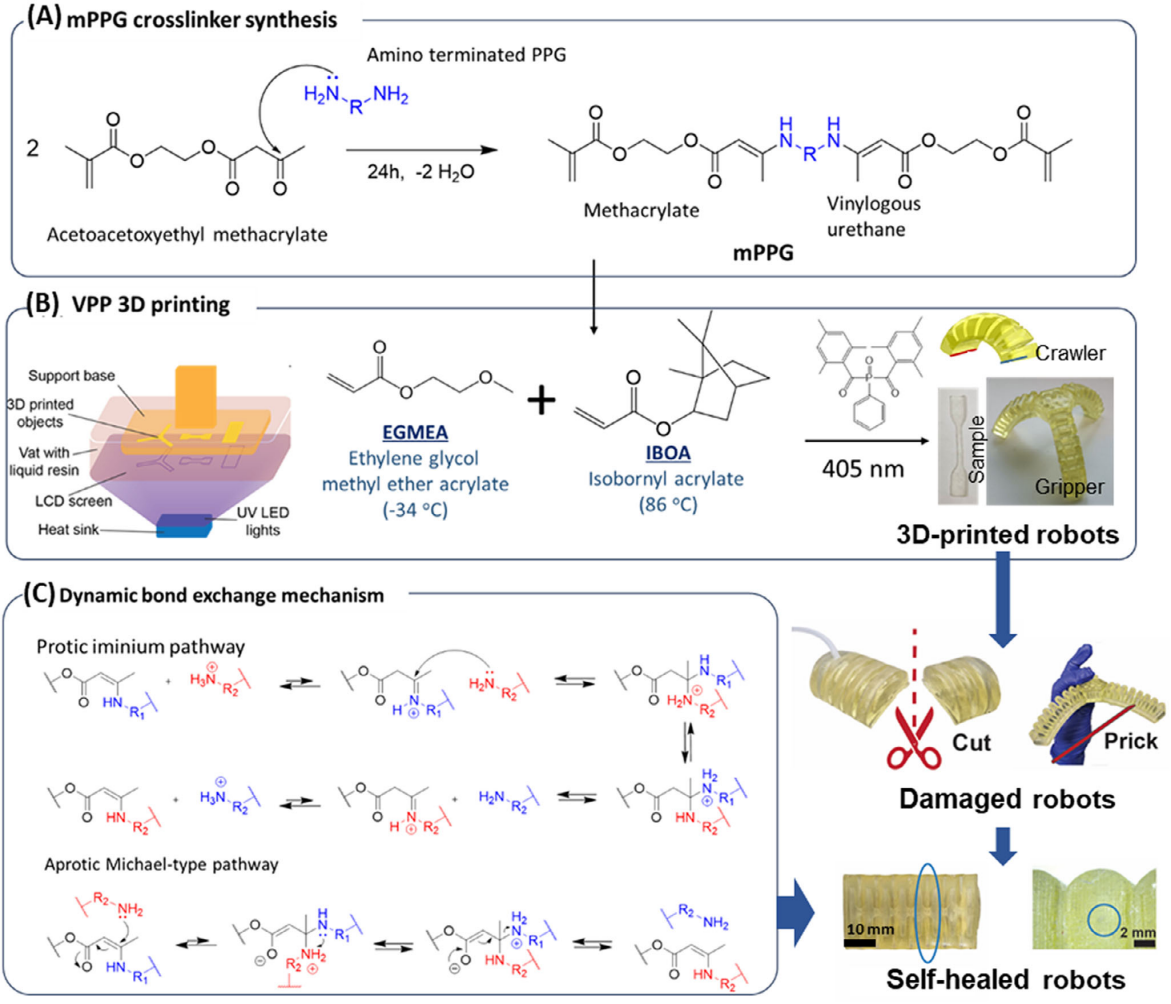
3D printing process, self‐healing mechanism, and 3D‐printed self‐healing robots. A) Reaction scheme of one‐pot one‐step crosslinker synthesis. Amine and acetoacetate condense to produce a vinylogous urethane moiety. R indicates the poly(propylene glycol), PPG, polymer backbone. B) Vat photopolymerization 3D printing process, where the vinylogous urethane‐modified polypropylene glycol (mPPG) crosslinker and photoinitiator (IBPO) are mixed with two monomers (EGMEA/IBOA = 1/1 molar ratio) and polymerized and printed onto the printing head layer‐by‐layer under UV wavelength λ = 405 nm. C) Dynamic bond exchange and self‐healing mechanism. Vinylogous urethane transamination pathways: protic iminium pathway or aprotic Michael‐type pathway.^[^ ^40^ ^]^

We synthesized and fabricated self‐healing soft grippers and crawlers by applying a solvent‐free vat photopolymerization (VPP) 3D printing process. The material is designed to be: 1) a polymer with a glass transition temperature (*T_g_*) below room temperature to obtain a soft material while maintaining mechanical strength; this was achieved by stoichiometrically mixing isobornyol acrylate (IBOA, *T_g_* = 86 °C) and ethylene glycol methyl ether acrylate (EGMEA, *T_g_* = ‐34 °C); 2) self‐healing by employing a vinylogous urethanes crosslinker to allow reprocessing at elevated temperature via reversible enamine exchange. A detailed outline of the formulations is discussed in Tables S1 and S2 (Supporting Information), and the composition is summarized in Table S2 (Supporting Information). A commercial 3D printer (Elegoo Saturn 2 and Nova 3D) was used for VPP 3D printing, where the covalently crosslinked elastomer objects are built layer‐by‐layer onto the moving metal substrate during the VPP 3D printing process (**Figure** **1**A). The selection of the monomer types, crosslinker molecular weight, formation, and printing parameters directly affect the structural stability and physical properties of the elastomer objects. The printed elastomer objects were post‐cured at 35 °C for 30 min under UV light prior to characterization.

The post‐cured grippers and crawlers are designed to be actuated by a pressure supply (0–16 kPa). With the introduction of damage, which includes the introduction of punctures and being cut into two halves, the robots can recover their functionality and initial performance after being self‐healed at room temperature for 24 h. After the end of their operational lifetime, the soft robots can be reprocessed for reuse (see Figure 3C), thereby reducing material waste and contributing to sustainable production.

Vinylous urethane crosslinker was synthesized by reacting primary *α*, *ω*‐diamine‐terminated poly (propylene glycol) (PPG) with different molecular weights (Jeffamine D230, D2000 or D4000) with acetoacetoxyethyl methacrylate (AAEM) in a *one‐pot one‐step synthesis*. The product was characterized using ^1^H NMR (Figure S1, Supporting Information) and GPC (Figure S2, Supporting Information) to confirm the successful modification. The synthesized crosslinker, vinylogous urethane‐modified polypropylene glycol (mPPG), and photoinitiator (BAPO) are mixed with two monomers (EGMEA/IBOA = 1/1 molar ratio) for VPP 3D printing (Figure 1B). The reversible amine‐enamine exchange and self‐healing mechanism via the protic iminium pathway or aprotic Michael‐type pathway are shown in Figure 1C,^[^ ^40^ ^]^ where the covalent bond exchange is self‐catalyzed via transamination by amine (Figure S1, Supporting Information).

### 2.2 Mechanical and Thermal Properties of 3D Printing

**Figure 2:**
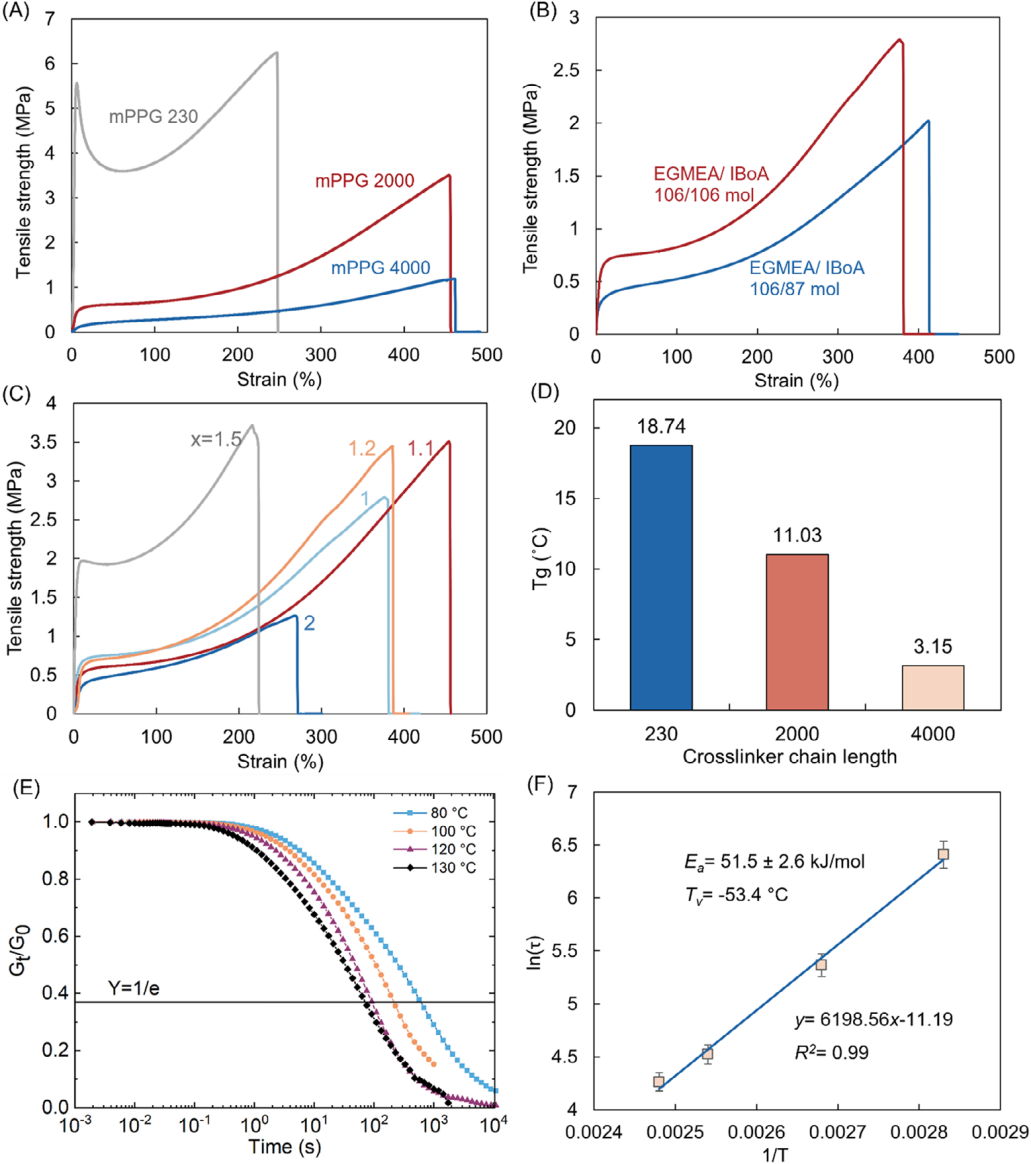
A) Tensile stress–strain curves of 3D‐printed specimens using a crosslinker with molecular weights at a ratio of 1:106:106 (mPPG/EGMEA/IBOA). B) Comparison of different monomer ratios (106:86 and 106:106 EGMEA/IBOA). C) Effect of varying mPPG 2000 crosslinker content (1, 1.1, 1.2, 1.5, and 2:106:106 mPPG/EGMEA/IBOA; see formulations in Table S2, Supporting Information). All samples were printed under identical conditions. D) Glass transition temperature (*T*_g_) of 3D‐printed polymers with different molecular weights (2000, 230, and 4000, respectively). E) Normalized stress relaxation profiles of the Formulation 2 between 80 and 130 °C. F) Arrhenius plot of ln(*τ*) versus inverse of temperature.

The printing condition has been optimized to be an exposure time of 5 s and a layer thickness of 25 µm based on our previous study.^[^ ^41^ ^]^ Tensile specimens were printed for mechanical evaluation according to ISO 527. **Figure** **2**A compares the effect of crosslinker mPPG with three different molecular weights (230, 2000, and 4000) on the mechanical properties of the printed parts. This shows that the crosslinked elastomers exhibit a high Young's modulus and is followed by yielding and plastic behavior when crosslinked with the shorter mPPG 230 molecular weight crosslinker; the material behaves more elastically when the mPPG crosslinker molecular weight increases to 2000 and 4000. We selected the mPPG 2000 crosslinker for further optimization due to its high elongation to failure and moderate tensile strength, thereby maintaining its structural integrity.

The monomers and crosslinker molar ratio are tailored to attain optimum mechanical properties. Molar ratios of 55:45 and 50:50 mol mol^−1^ of EGMEA/IBOA monomer ratios were tested. The tensile strength and elongation at break were 2.75 MPa and 380% for 55:45, respectively, and 2.01 MPa and 415% for 50:50, respectively (Figure 2B). The molar ratio of the mPPG crosslinker was also varied with of mPPG/EGMEA/IBOA ratio of (1‐2):106:106 at a fixed EGMEA/IBOA monomer ratio of 50:50 mol mol^−1^. It was observed that 1.1:106:106 (Formulation 2 in Table S2, Supporting Information) provided the best balance of elastomer mechanical properties with a tensile strength of 3.51 MPa and elongation at break of 454% (Figure 2C).

Figure 2D shows the glass transition temperature (*T*_g_) of 3D‐printed polymers with different crosslinker molecular weights. The *T_g_* was measured using differential scanning calorimetry DSC, where a *T_g_* lower than the operation temperature is crucial to ensure high segmental mobility to attain soft materials and self‐healing properties. The *T_g_* of 3D printed objects using different mPPG crosslinker molecular weights decreased from 18.7 °C, 11.0 to 3.2 °C as the mPPG crosslinker molecular weight increased from 230, 2000 to 4000. This is due to the larger mass fraction of PPG (*T_g_* = ‐73 °C) in the material, at the same molar fraction of crosslinker, which plasticizes the resultant polymer network. The formulation of mPPG/EGMEA/IBOA = 1.1/106/106 with the PPG spacer length of 2000 g mol^−1^ was chosen to offer the optimum combination of tensile strength and elongation at break (Formulation 2, Table S2, Supporting Information).

### 2.3 Dynamic Network and Self‐Healing Mechanism

The dynamic covalent network functionality arises from the vinylogous urethane moiety. The reversible amine‐enamine exchange and self‐healing mechanism via the protic iminium pathway or aprotic Michael‐type pathway are shown in Figure 1C,^[^ ^40^ ^]^ where the covalent bond exchange is self‐catalyzed via transamination by amine (Figure S1, Supporting Information). The embedded dynamic vinylogous urethane bonds allow the 3D‐printed elastomer objects to self‐heal when subject to a thermal trigger, thus recovering their structural integrity and physical properties.

Shear stress relaxation tests were conducted between 80 and 130 °C (Figure 2E) to monitor the decay of modulus over time, indicating the dissipation of the applied stress by the relaxation. An Arrhenius plot of the characteristic relaxation time versus the inverse of temperature (Equation (1)) was fitted to demonstrate the effect of temperature on the relaxation time (Figure 2F).

(1) $$\ln\left(\tau \right)=\frac{E_{a}}{RT}+\ln\left(\tau_{0}\right)$$

where the characteristic stress relaxation time τ refers to the time taken when the relaxation modulus decreases to 0.37 (1/e) of the initial modulus as a reference point for comparison, R is the gas constant, and *T* is the Kelvin temperature. The terms *τ*_0_ and *E_a_* are the fitting parameters of the apparent relaxation time at infinite temperature and the energy term, respectively.

The apparent activation energy (*E_a_*) is the energy required to allow relaxation upon applied stress, as shown in Figure 2F. The calculated *E_a_* = 51.5 kJ mol^−1^ lies within the other reported values of 20 – 70 kJ mol^−1^ for an enamine vitrimer.^[^ ^42^, ^43^, ^44^, ^45^ ^]^ According to the Arrhenius fitting, the relaxation time can be extrapolated to 30 °C, which is the operation temperature of later self‐healing experiments. The theoretical 1/e relaxation time at 40 °C is calculated to be 10 586.1 s (2.94 h), and the theoretical 95% relaxation time is 11.76 h. Experimental measurement has been attempted despite prolonged measurement time and partial slippage at lower temperatures. However, the measured τ is 27 705 s (7.7 h), 2.6 times longer than the theoretical value. This is expected that multiple relaxation mechanisms are complexed at lower temperatures and therefore it deviates from the Arrhenius relationship (Figure S4, Supporting Information). This is further demonstrated in the recovery of tensile properties.

**Figure 3:**
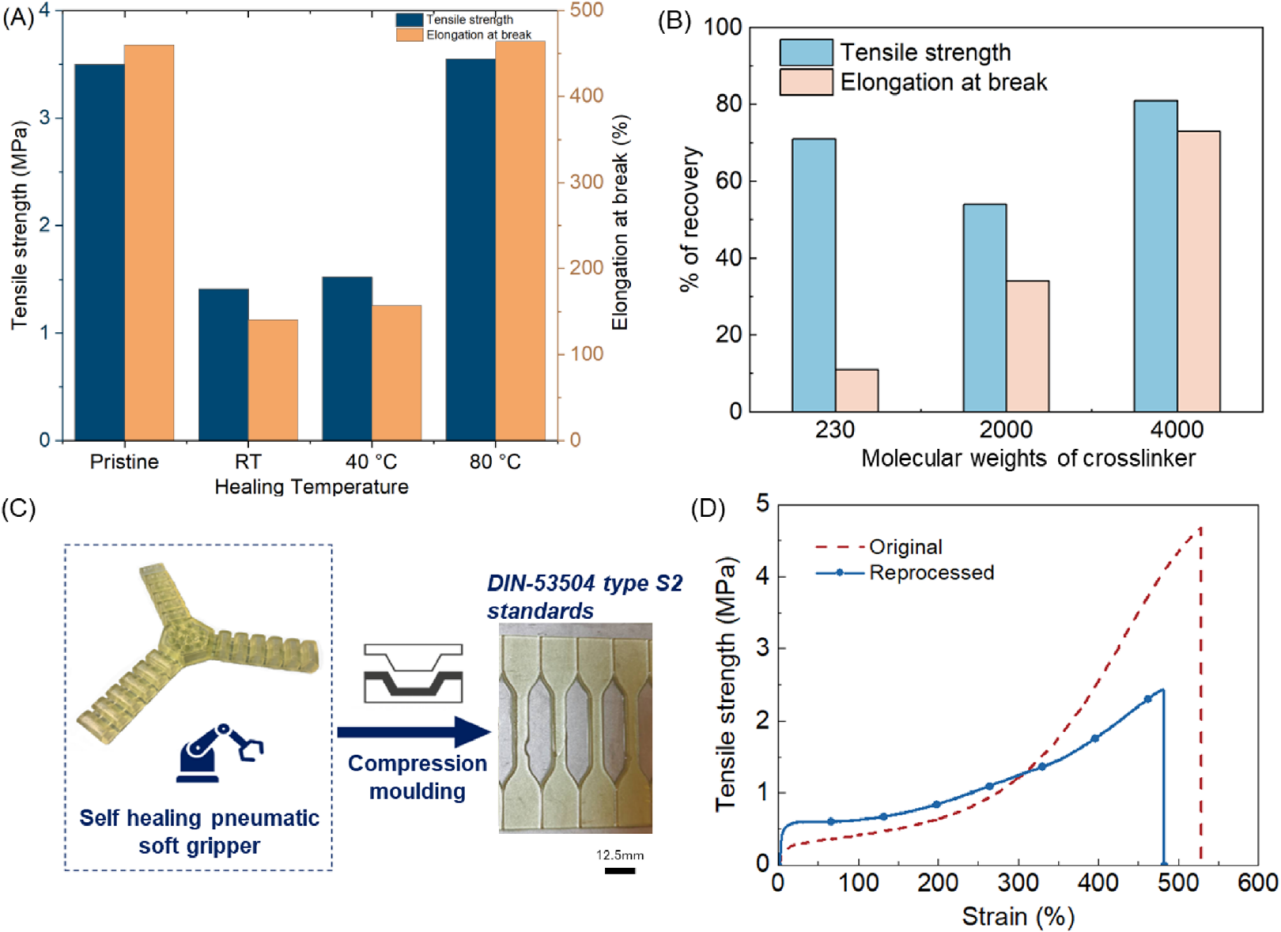
A) Tensile tests of pristine Formulation 2 and after healing at 25, 40, and 80 °C for 24 h. B) Percentage of recovery of Formulations 2, 6, and 7 (Table S2, Supporting Information) using a crosslinker with different molecular weights (2000, 230, and 4000, respectively) after healing at 40 °C for 48 h. C) Schematic of reprocessing the 3D printed part into other objects via compression molding. D) Tensile tests of pristine 3D printing and after reprocessing via compression molding at 150 °C for 1 h.

Self‐healing tests were conducted on the tensile specimens half‐cut and healed at 25, 40, and 80 °C for 24 h, respectively. **Figure** **3**A shows the tensile strength and elongation at break of the samples with mPPG 2000 crosslinker. At room temperature or 40 °C, the recovery is moderate at 40% and 43% for tensile strength and 31% and 34% for elongation, respectively. This agrees with the slow stress relaxation at room temperature. At 80 °C, full recovery was observed for both tensile strength and elongation. Figure 3B shows the recovery percentage of 3D printed samples with different crosslinker molecular weights and healed at 40 °C for 48 h. The recovery of elongation at break increases with the increase of the crosslinker molecular weight due to the higher segmental mobility of the polymer network, as indicated by the lower *T_g_* values. The tensile strength and elongation at break of the sample cured with mPPG 4000 recovered up to 81% and 73%, respectively.

To assess the thermal processability of the printed elastomers, the printed material (Formulation 2 was used here, Table S2, Supporting Information) was subject to cryo‐grinding into powders and then compression molding at 150 °C for one hour into the desired geometry (Figure 3C). Mechanical properties of reprocessed elastomers are compared to the originally printed samples (Figure 3D) and show a good recovery with a tensile strength of 2.4 MPa and elongation at break of 480.1%.

### 2.4 3D‐Printed Self‐Healable Gripper

#### 2.4.1 Design and Demonstration

**Figure 4:**
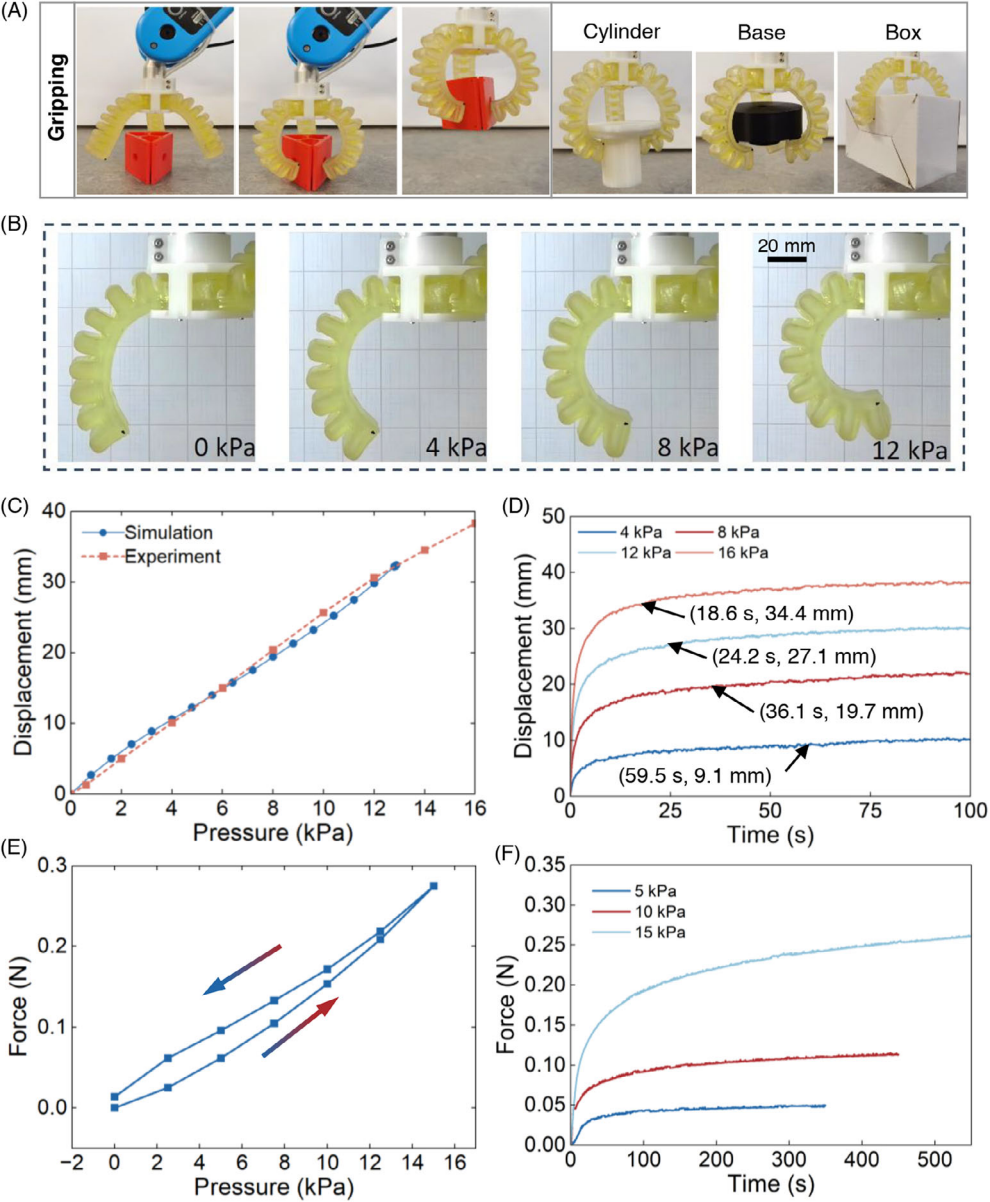
Mechanical characteristics of the 3D‐printed self‐healable gripper. A) Gripping function demonstration. B) Displacement state under varying pressure conditions. C) Horizontal displacement static results. D) Horizontal displacement dynamic results. E) Static force results. F) Dynamic force results.

Soft actuators and robots are usually pre‐designed and fabricated by conventional molding to achieve a variety of deformations/motions such as bending, extension, contraction, and twisting. The soft actuators or robots are prone to punctures or cuts from sharp items, and their fabrication requires the design and production of molds through 3D‐printing, molding, and curing of parts, and assembling using adhesives for final products. By implementing self‐healing and 3D‐printing capabilities on soft robots, their ability to withstand unforeseen damage can be greatly improved, and the manufacturing process can be streamlined. Based on the materials optimization outlined earlier, a fully 3D‐printable and self‐healing gripper was designed and 3D‐printed based on Formulation 2 (Table S2, Supporting Information) as a demonstration. The gripper consisted of three soft pneumatically‐driven finger‐shaped actuators, as shown in **Figure** **4**A and Figure S5A (Supporting Information). The designed gripper was simulated in Ansys/Workbench (see Figure S7A, Supporting Information), and the gripping function is realized by pressurizing the three actuators simultaneously to grip objects with different shapes, as shown in Figure 4A and Movie S1 (Supporting Information).

#### 2.4.2 Mechanical Characterization

The mechanical characterization of the gripper was conducted using the experimental setups (Figures S8 and S9, Supporting Information) for deformation displacement and the tip force of the gripper finger. The characteristics, including static and dynamic displacement and force, are presented in Figure 4B–F. The horizontal displacement state, as shown in Figure 4B, was captured by a camera and tracked in an open‐source software Kinovea, using marking points on the gripper and crawler as reference points. As shown in Figure 4C, the horizontal displacement increases almost linearly with the supply pressure, reaching a maximum of 38.3 mm at 16 kPa. Figure 4D shows that the gripper deforms to 90% of the steady–state deformation within 20–60 s for dynamic displacement responses at 4, 8, 12, and 16 kPa, gradually increasing to the steady‐state value. The single finger can achieve a maximum force of 0.27 N in the static force investigations, with hysteresis inherited from the polymers, as shown in Figure 4E. For the dynamic force test results in Figure 4F, the tip force varies similarly to the displacement when pressurized. This takes more than 300 s to reach a steady state, and the settling time increases with the driving pressure. This delay may be due to the creep behavior of the polymer caused by viscosity, as seen in Figure S5 (Supporting Information), in which the material deforms slowly with time at a constant pressure, resulting in an increase in tip force.

### 2.5 3D‐Printed Self‐Healing Crawler

#### 2.5.1 Design and Mechanical Characterization

**Figure 5:**
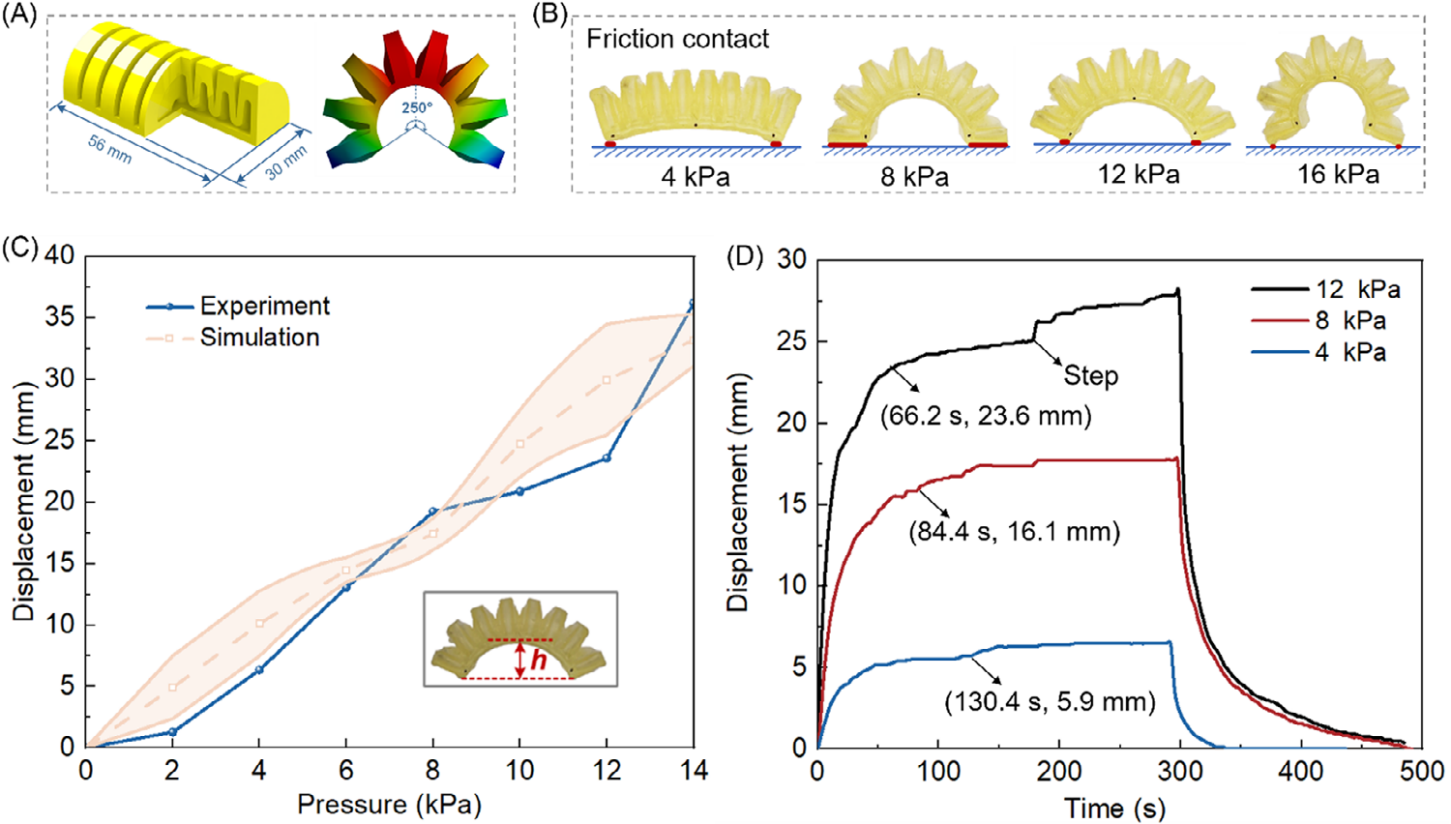
Design and mechanical characterization of the 3D‐printed self‐healable crawler. A) Design and FEM simulation. B) Friction contact changes at bending deformation. C) Static vertical deformation. D) Dynamic vertical deformation.

Advanced soft robots that can perform multiple functions are highly desirable. A crawler with complex inner chambers has been designed and 3D‐printed, as shown in **Figure** **5**A. When pressurized, the crawler can bend from its original flat status to 250°. A model of the crawler's deformation was created using Ansys/Workbench (see Figure S7, Supporting Information) and validated through experiments. The 3D‐printed crawler was characterized by both static and dynamic displacement tests of the crawler in Figure 5C,D. Figure 5C shows the crawler's vertical displacement measurement, and the simulation follows the experimental result well, in which the vertical displacement reached a maximum of 36.2 mm at 14 kPa. The results revealed that when the pressure was between 0–8 kPa, the crawler experienced almost linear deformation, while the deformation became nonlinear at higher pressures. When the crawler was subjected to supply pressures of 4, 8, and 12 kPa, it achieved vertical displacements of 5.9, 16.1, and 23.6 mm in 130, 84, and 66 s, respectively. A step increase was observed at 177 s of the 12 kPa curve because the friction force between the crawler and the ground suddenly changes during the bending deformation. The friction force change is because the contact points between the ground and the crawler change with increased pressure, as shown in Figure 5B.

#### 2.5.2 Locomotion and Dynamic Response

**Figure 6:**
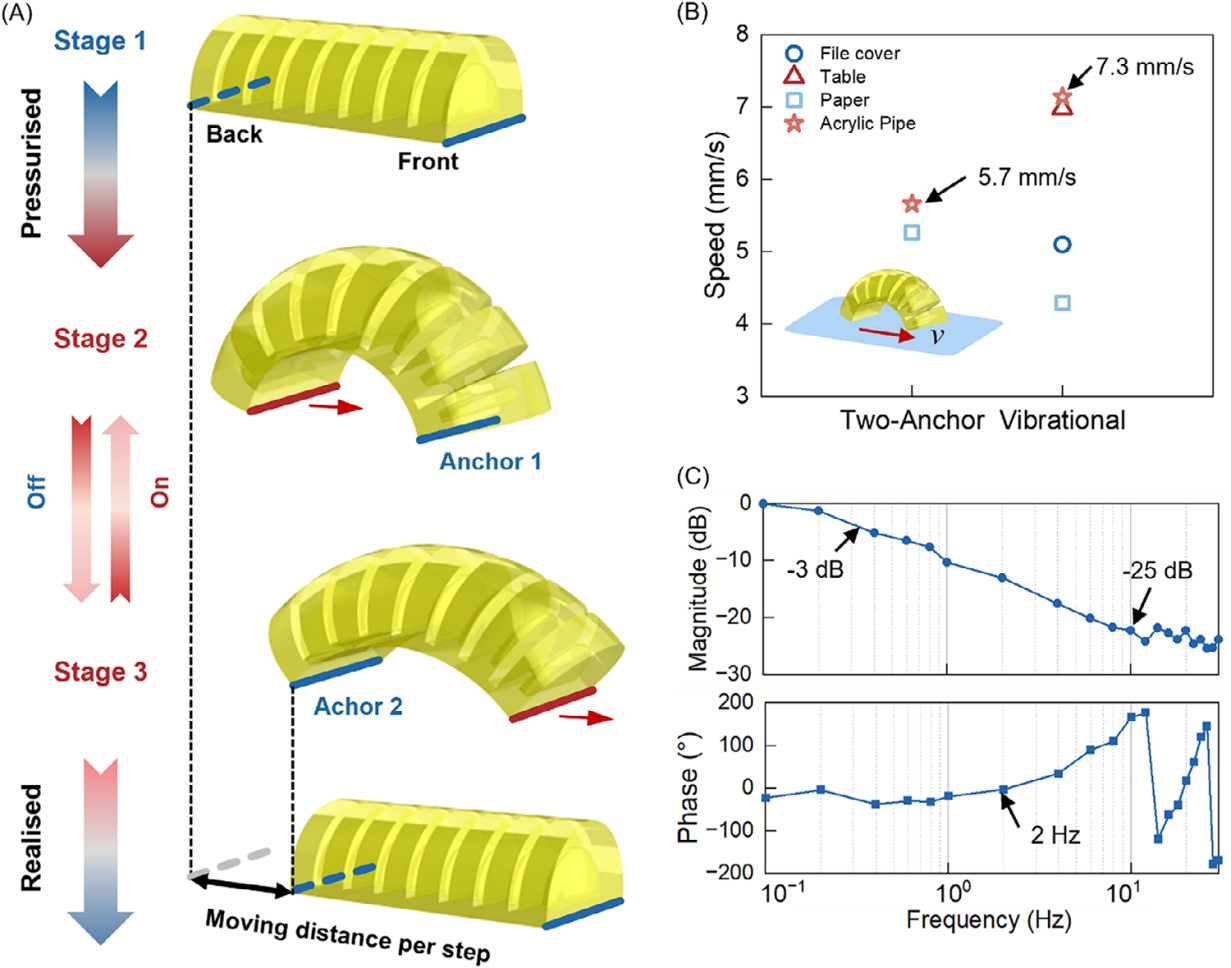
Locomotion and frequency response of the 3D‐printed self‐healable crawler. A) Crawling mechanism. B) Crawling speed on different surfaces. C) Frequency response.

The crawler is able to crawl by periodically activating the pressure supply, as shown in Movie S2 (Supporting Information). Under pressure, the crawler undergoes two stages of deformation. During Stage 1, it is in its original state. When the pressure increases, it moves to Stage 2, where the back end moves forward while the front end acts as Anchor 1, as shown in **Figure** **6**A, due to the greater friction generated from the front end. When the pressure is released, the crawler returns to a lower position in Stage 3, where the ground contact points of the front and back ends change so that the back end acts as Anchor 2 due to greater friction while the front end moves forward in Stage 3. As a result, the crawler moves forward by a step distance after Stage 2 and Stage 3, and this mode is termed a “two‐anchor mode”. The crawler is designed to move forward continuously through a periodic pulse width modulation (PWM) on/off signal to the supply pressure. This process cycles between Stages 2 and 3. When the supply pressure is removed and fully released, the crawler stops and returns to its original status at Stage 4. The crawling speed can be adjusted by varying the pulse width, amplitude, and frequency of the PWM signal. As the frequency increases, the crawler can deform quickly with low amplitude, thus moving in a “vibrational mode”, as shown in Movie S3 (Supporting Information). To investigate the material's friction coefficient effect on the motion of a crawler, we conducted speed tests during two crawling modes – two‐anchor mode and vibrational mode – on surfaces with varying friction coefficients as shown in Figure S9C (Supporting Information). Figure 6B shows that maximum speeds of 5.7 and 7.3 mm s^−1^ were achieved with two‐anchor and vibrational modes on an acrylic pipe, respectively, due to the maximum friction coefficient of 0.64 (see Figure S9D, Supporting Information). The frequency domain analysis was conducted on the system, including the crawler with the pressure control system, with a frequency range of 0.1–30 Hz. The bandwidth is ≈0.3 Hz (−3 dB), and the magnitude ratio decreases to −25 dB at 10 Hz and above, while the phase difference remains close to 0 until 2 Hz, as shown in Figure 6C.

### 2.6 Self‐Healing Validation of Gripper and Crawler

**Figure 7:**
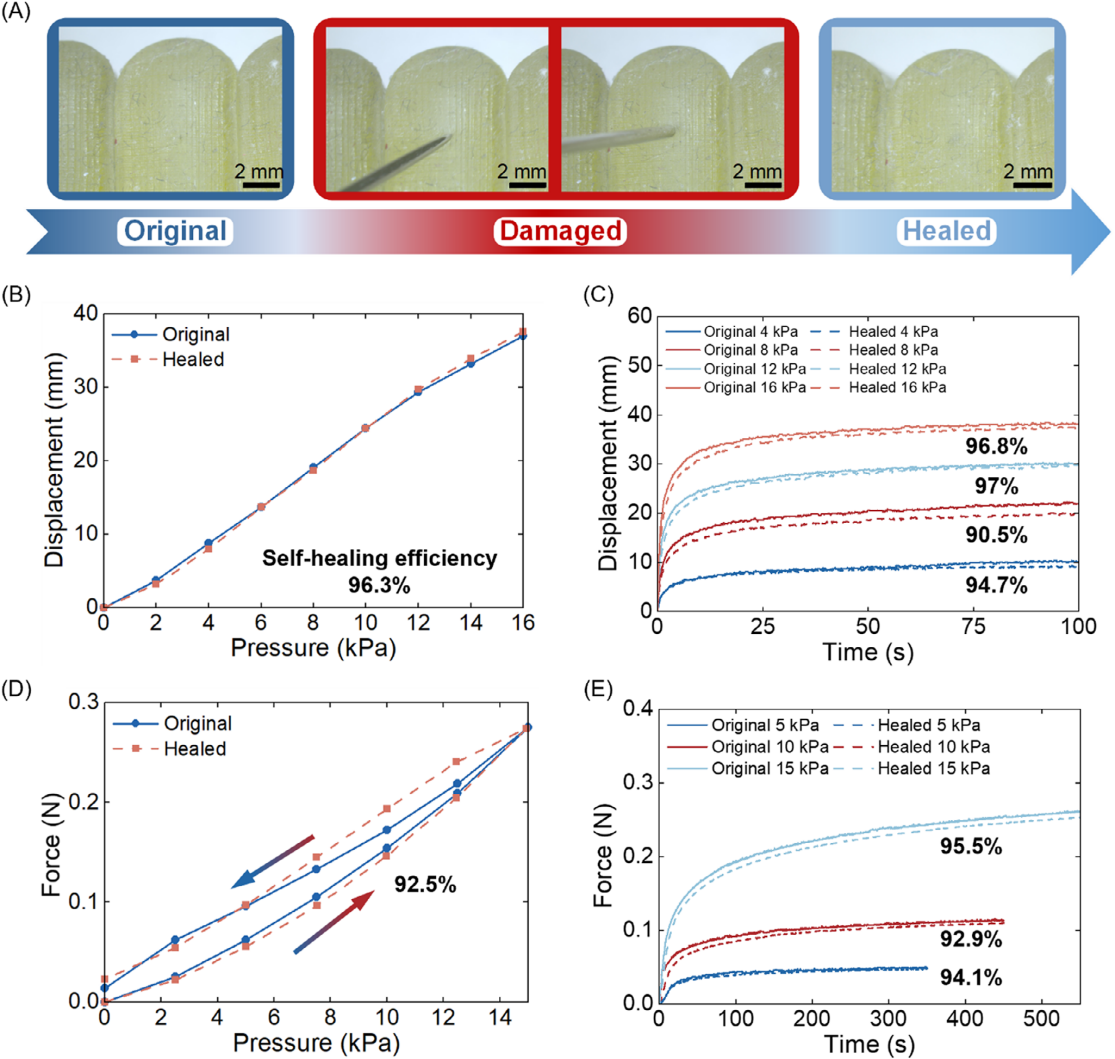
Self‐healing validation of the 3D‐printed self‐healable gripper. A) Original, damaged (punctured), and healed photos from the microscope. B) Static displacement. C) Dynamic displacement. D) Static force. E) Dynamic force.

The self‐healing properties of the soft gripper were examined by puncturing the gripper and healing at room temperature (25 °C) for 24 h. **Figure** **7**A shows the original, damaged, and healed photos under an optical microscope. The gripper completely healed and exhibited exceptional self‐healing capability at room temperature. In Figure 7B–E, we compared the mechanical properties of the original and healed gripper, and only slight differences were observed. The static and dynamic displacements tests exhibit high self‐healing efficiencies of 96.3%, 94.7% (4 kPa), 90.5% (8 kPa), 97% (12 kPa), 96.8% (16 kPa), and static and dynamic force tests with healing efficiencies of 92.5%, 94.1% (5 kPa), 92.9% (10 kPa), and 95.5% (15 kPa), respectively. The average self‐healing efficiency of the gripper reaches 94.5%. Details of the calculated self‐healing efficiencies are outlined in Figure S10 (Supporting Information).

**Figure 8:**
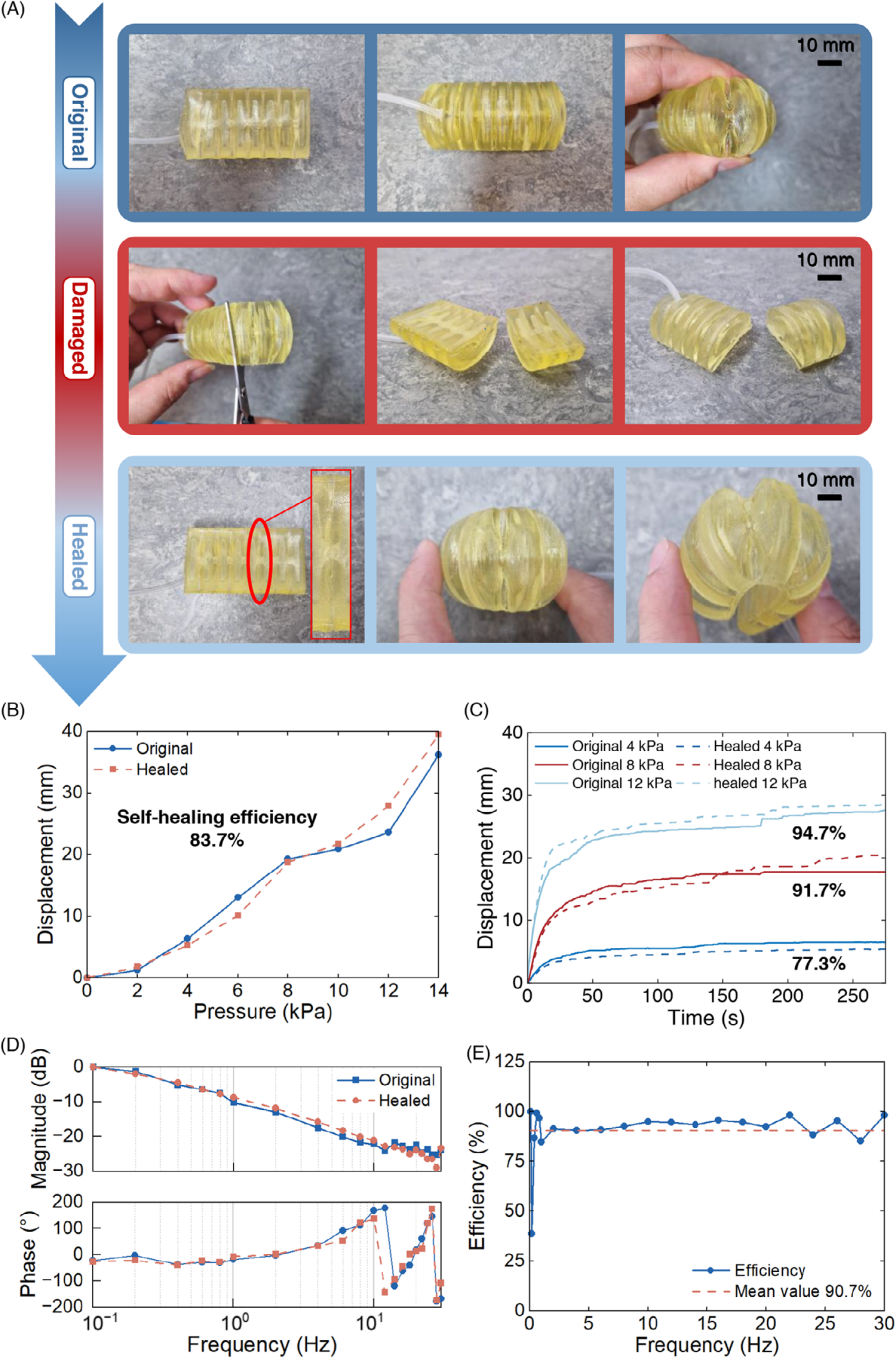
Self‐healing validation of the 3D‐printed self‐healable crawler. A) Original, damaged, and healed photos. B) Static displacement. C) Dynamic displacement. D) Frequency response test. E) Average self‐healing efficiency of frequency response magnitude.

To investigate the effects of the introduction of more severe damage, the crawler was cut in half, and the failure surfaces were subsequently pressed together and healed at room temperature (25 °C), as shown in **Figure** **8**A. The crawler self‐healed with an almost invisible seam at the base of the cut location, showing excellent self‐healing ability after the introduction of significant damage. Figure 8B–E compares the mechanical characteristics of the original and healed crawler. The static displacements show only an average below 2% difference between the original and healed crawler, with a self‐healing efficiency of 83.7%. The dynamic performances of the crawler are well recovered with self‐healing efficiencies of 77.3% (4 kPa), 91.7% (8 kPa), 94.1% (12 kPa), and 90.7% in terms of the frequency response. The average self‐healing efficiency of the crawler reaches 87.5%, and the detailed calculation of self‐healing efficiencies is presented in Figure S11 (Supporting Information). The crawler motions before and after healing in Movie S4 (Supporting Information) evidence that the motion capability has been fully recovered. The excellent self‐healing ability and the easy healing approach can significantly increase the lifetime and improve the robustness of the soft robots. When the soft robot is damaged, it can quickly heal at room temperature by attaching the damaged surfaces.

## 3 Discussion and Conclusion

Soft robots with an inherently high compliance have the potential to provide adaptivity and resilience for uncertain and complex environments, safe human‐machine interactions, and complex motions. Since high compliance also results in vulnerability to sharp objects and damage, soft robots with self‐healing functionality are desired. The majority of soft robotic devices are currently fabricated by conventional molding and casting, which is time‐consuming and has limitations on geometric design. Here, we have successfully incorporated additive manufacturing and self‐healing into soft pneumatic robots. Vat photopolymerization (VPP) polymers with reversible dynamic vinylogous urethane bonds were formulated, synthesized, and used to create complex geometry soft self‐healable pneumatic grippers and crawlers via additive manufacture.

The polymerization of the formulated polymer can be initiated through free‐radical polymerization (FRP) using UV light. As a result, they can be 3D‐printed without adaptation on commercially available vat photopolymerization‐based 3D printers, including stereolithography (SLA), digital light processing (DLP), and liquid crystal display (LCD) printers. The polymer formulation and 3D‐printing parameters, including post‐curing conditions, were optimized to achieve a combination of high tensile strength of 3.51 ± 0.1 MPa and elongation at break of 454 ± 56%, coupled with high self‐healing performance with maximum recovery rates of 83% and 71% on 3D‐printed tensile test samples. We evaluated four different crosslinker molecular weights (230, 2000, 4000), five different crosslinker molar ratios (1, 1.1, 1.2, 1.5, and 2), two layer thicknesses (30 and 50 µm), two different layer exposure times (3.75 and 5 s) and four different post‐curing conditions (30 or 60 min at 35 or 60 °C); many other variations could be chosen to tune the material mechanical properties according to the requirement of specific applications.

Based on the optimal formulation and processing conditions, a soft gripper featuring three fingers and 24 air chambers, as well as a crawler with complex chambers, was designed and 3D‐printed. The FEM simulation and experimental mechanical characterization were conducted to evaluate their mechanical performance. The single finger of the gripper achieves a maximum force of 0.27 N, and the crawler achieves a maximum crawling speed of 7.3 mm s^−1^. The gripper and crawler were punctured, cut into two halves, and allowed to self‐heal at room temperature for 24 h without external stimuli to examine their self‐healing capability. We compared mechanical performance, including the static and dynamic deformation and force tests, and frequency responses of the original and healed robots. The gripper and crawler achieved excellent self‐healing efficiencies of 94.5% and 87.5%, respectively, which validated the feasibility and advantages of additive manufacturing soft self‐healable robots with complex geometry designs. In addition to the geometries produced in the paper, the vat photopolymerization printing process can be used to produce a variety of geometries of relevance to soft robotics, such as soft actuators with intricate air channels, wearable sensor substrates, or bio‐inspired structures that include fin‐like components for swimming robots.

The healing efficiency metrics adopted in this work were designed to comprehensively reflect the functional recovery of soft robots, including mechanical properties (e.g., tensile strength and elongation at break) of materials and static/dynamic performance indicators directly linked to their operational functions. This multi‐indicator approach can avoid potential biases associated with single metrics. For example, a material with high tensile strength recovery may still fail to restore functional performance (e.g., insufficient gripping force) if the dynamic response of the robot's air chambers is not recovered. The healing efficiencies (94.5% for the gripper, 87.5% for the crawler) were calculated by averaging the efficiencies of all functional indicators, ensuring a holistic assessment of healing effectiveness.

To evaluate the reprocessability and recyclability, the 3D‐printed grippers were cryogrind and compression molded into tensile specimens at 150 °C for 1 h. The tensile performance of the reprocessed material showed tensile strength of 2.4 MPa and elongation at break of 480%, demonstrating good reprocessability due to the vitrimer chemistry, thus reducing the materials waste and contributing to sustainable manufacturing of soft devices.

We compared the mechanical properties, self‐healing ability, and reprocessability of the elastomer in this work with those of other refs. [46, 47, 48, 49] in Table S4 (Supporting Information). Our VPP‐compatible elastomer enables high‐resolution (25 µm) printing of complex geometries, while elastomers reported in the literature to date rely on solution casting or molding. The printed gripper and crawler heal at room temperature, exhibiting excellent recovery of robotic functions, a critical requirement for real‐world applications.

Despite the excellent room temperature self‐healing and complex geometry manufacturing ability of our approach, the 24 h window may be impractical for time‐sensitive use cases (e.g., emergency search‐and‐rescue, real‐time industrial fault repair). External stimuli such as heating and light could be promising solutions. Moderate temperature increments can effectively accelerate healing without compromising efficiency due to the reduced relaxation time of the dynamic network and faster bond rearrangement. For other applications or operating conditions, the selection of other monomers to tailor the *T_g_* and mechanical properties, as well as the type of reversible chemistry to dictate the self‐healing/reprocessing temperature, can be optimized.

Light‐induced catalysis could be achieved by incorporating photoacid generators (PAGs) into the polymer matrix. Vinylogous urethane bond exchange is known to be autocatalyzed by protons; upon exposure to low‐intensity UV light (e.g., 405 nm, compatible with our 3D printing wavelength), PAGs would release protons to accelerate transamination reactions. Our current approach uses commercial vat photopolymerization printers, which is suitable for prototyping and small‐scale robots but limits its scalability to meter‐scale robots or high‐volume production. Modular printing and self‐healing assembly could be future pathways for increasing scalability by shifting from monolithic printing to printing small, standardized submodules that can be self‐healed into large robots.

In summary, vat photopolymerization (VPP) polymers with reversible dynamic vinylogous urethane bonds were formulated, synthesized, and used to create complex geometry and damage‐tolerant soft self‐healable pneumatic grippers and crawlers that can be reprocessed and reused. The static and dynamic performance of the soft robots was investigated in detail after being punctured, cut in half, and left to self‐heal at room temperature for 24 h, achieving excellent self‐healing efficiency close to 90% in terms of both mechanical static and dynamic performance. Our approach therefore addresses a critical limitation of existing self‐healing soft robots, which typically require some form of additional external stimuli (e.g., light, heat, or magnetic field). The ability to combine additive manufacturing, efficient room‐temperature self‐healing, and a capability to be reprocessed provides a new route to create customizable, robust, resilient, and recyclable soft robots. Potential applications include safe human interaction, wearable devices, medical rehabilitation, bio‐inspired exploration, adaptive grippers for manufacturing, and disaster response scenarios.

## 4 Experimental Section

### Materials

PPGwith four different molecular weights (g/mol), i.e., PPG 230 (Jeffamine D‐230), PPG 2000 (Jeffamine D‐2000), and PPG 4000 (Jeffamine D‐4000), were purchased from Hunstman Corporation. Acetoacetoxyethyl methacrylate (AAEM) was used to functionalize the PPG to make dual‐functionalized crosslinkers. IBOAEGMEA were used as monomers. Phenylbis (2,4,6trimethylbenzoyl) phosphine oxide (BAPO) was selected as a photoinitiator. All the chemicals were purchased from Sigma Aldrich and used as received to formulate the polymer, as shown in Table S1 (Supporting Information).

### Synthesis of Vinylogous Urethane‐Modified Cross‐linkers

mPPG was synthesized by the reaction of AAEM with PPG as shown in Figure 1A. PPG was dispersed in isopropanol in a weight ratio of 2:1 cooled in an ice bath and stirred magnetically until the contents became clear. AAEMwas added dropwise and then stirred at room temperature for 24 h. The solvent and residual water were removed by vacuum distillation at 40 °C. A series of mPPG were synthesized based on the formulations shown in Table S2 (Supporting Information). The resulting crosslinkers were sealed and stored in the dark at ambient conditions.

### Vat photopolymerization (VPP) 3D‐printing

The default printing parameters of Nova 3D and Elegoo Saturn 2 printers were used to convert imported files into printer files in Lychee Slicer (version 5.1.8). The exposure times for the burn‐in and normal layers were set to 5 and 3.75 s and the layer thickness was set to 25 and 50 µm. mPPG EGMEA IBO ABAPO were stoichiometrically mixed and kept at room temperature of 25 °C for 1 h before being placed into the vat of the 3D printers. Tensile test samples, grippers, and crawlers were printed under the same conditions using an Elegoo Saturn 2 3D printer (λ = 405 nm). The distance between the LCD screen and the printing substrate was regulated and calibrated at the start of each printing session. At the end of the printing, the printed objects were removed from the substrate and soaked in isopropanol for 1 h to remove the extra monomers. A syringe was used to rinse the inside chambers through the air inlets of the printed soft robots with isopropanol before they were post‐cured for 30 min in Formlab Form Cure (λ = 405 nm).

### Material Characterization

Proton nuclear magnetic resonance (^1^H NMR) spectra were obtained using a Bruker HD‐400 spectrometer in CDCl_3_. The chemical shifts were reported in parts per million (ppm) relative to residual solvent peaks.

Gel permeation chromatography (GPC) measurements were conducted using an Agilent 1260 Infinity II MDS instrument equipped with a differential refractive index (DRI) detector and a viscometer. The system was equipped with two PLgel Mixed C columns (300 × 7.5 mm) and a PLgel 5 µm guard column. Chloroform with a 2% triethylamine (TEA) additive and ethanol as a flow rate marker were used as the eluent. The samples were run at a flow rate of 1 ml min^−1^ at a temperature of 30 °C. Polymethyl methacrylate (PMMA) was used for calibration on Agilent EasyVials. Before injection, the analyte samples were filtered through a GVHB membrane with a pore size of 0.22 µm. The number averaged and peak molecular weight (*M_n_*, *M_p_*) were determined using Agilent GPC/SEC software with PMMA calibration.

### Mechanical Characterization

Tensile testing was performed using a Shimadzu EZ LX Universal Testing instrument according to DIN‐53504 type S2 standards. The extension rate for all tests was set at 200 mm min^−1^ with a 500 N load cell, and the tests were conducted at room temperature following ASTM‐D412 guidelines.

Cyclic stress testing was carried out for five cycles, and for each cycle the clamped specimen was stretched to an elongation of 100% and then retracted to its original position under a controlled extension rate of 50 mm min^−1^ without intervals. The hysteresis loss ratio between two different hysteresis loops can be calculated according to Equation (2),

(2) $$H_{i}=\frac{A_{i}}{A_{up}}$$

where *H_i_*, *A_up_* and *A_l_* are the hysteresis loss ratio, the area under the uploading stress‐strain curve, and the area of the loop curve, respectively, for the *i*_th_ cycle.

Shear stress relaxation tests were carried out between the temperatures of 80 and 130 °C using an Anton Paar rheometer MCR 302. A parallel plate geometry with a diameter of 25 mm was used, and the gap was set at 1 mm for all samples. The strain was 2.5%, which was in the linear viscoelastic regime, and to ensure contact between the sample and the plates, an axial force of 2.5 N was applied to the sample (Figure S5, Supporting Information).

Reprocessibility was evaluated by compression molding cyro‐grinded specimens with a hydraulic Rondol bench‐top lab press at 150 °C for 60 min at 30 kN closing force. Sample powder was loaded between the smooth polytetrafluoroethylene (PTFE) release liner and preheated for 3 min at 0.1 kN. Manual bumping was performed to evacuate the air from the sample by increasing the force by 5 kN each time, followed by release to 0 kN until the final closing force was reached. The sample was cooled to room temperature (RT) at 30 kN closing force.

The mechanical characterization tests of the 3D‐printed self‐healable gripper and crawler were conducted using the pressure control system (Figure S8, Supporting Information) and the force tests were conducted with the customized force measurement setup (Figure S9A, Supporting Information) using a customized force beam sensor.

The friction tests of the 3D‐printed self‐healable crawler on surfaces with different friction coefficients were conducted. The experimental setup in Figure S9C (Supporting Information) was used for measuring the friction coefficients of different surfaces, and the results were shown in Figure S9D (Supporting Information). The crawler was driven by a Pulse Width Modulated (PWM) pressure, with an amplitude of 50 kPa, a pulse width of 35%, and a frequency of 1 Hz for the two‐anchor mode and 15 kPa, 35%, and 10 Hz for the vibrational mode.

## Conflict of Interest

The authors declare no conflict of interest.

## Supporting information



Supplemental Movie 1

Supplemental Movie 1

Supplemental Movie 2

Supplemental Movie 3

Supplemental Movie 4

## Data Availability

The data that support the findings of this study are available from the corresponding author upon reasonable request.
