# Supplementary material for: Self‐Healing and Reprocessable Soft Robots Using 3D Digital Light Printing
Source: Adv Sci (Weinh). 2025 Nov 3;13(2):e16901. doi: 10.1002/advs.202516901 (PMC12786329; doi:10.1002/advs.202516901)
Supplement: Supplementary file 1 — Supplemental Movie 1 [file ADVS-13-e16901-s004.docx]

Supporting Information

Self-Healing and Reprocessable Soft Robots using 3D Digital Light Printing

Chenggang Yuan^1^, Yuqing Qin^2,3^, Miaomiao Liu^1^, Wai Hin Lee^2^, Mantas Drelingas^2,3^, Sebastian Fieldhouse^2^, Alan M. Wemyss^2^, Poh Sheng Tay^1^, David M. Haddleton^3^, Chris Bowen^1^, Chaoying Wan^2^*, Min Pan^1^*

^1^Department of Mechanical Engineering, University of Bath, UK.

^2^International Institute for Nanocomposites Manufacturing (IINM), University of Warwick, CV4 7AL, UK.

^3^Department of Chemistry, University of Warwick, CV4 7AL, UK.

The PDF file includes:

Figures S1 to S11

Tables S1 to S4

Other Supplementary Material for this manuscript includes the following:

Movies S1 to S4

**Proton nuclear magnetic resonance**

Proton nuclear magnetic resonance (^1^H NMR) spectra were obtained using a Bruker HD-400 spectrometer in CDCl_3_. The chemical shifts were reported in parts per million (ppm) relative to residual solvent peaks.

Acetoacetoxyethyl methacrylate (AAEMA), poly (propylene glycol) (PPG) with a molecular weight of 2000 (33 repeating units of propylene glycol), and mPPG 2000 were characterized by proton nuclear magnetic resonance (^1^H NMR). **Figure S1** shows all carbon-based hydrogens in the spectra. The integration of hydrogen in the α location of the terminal amines, labelled *j* in Figure S1 (*δ* = 1.16 ppm) and hydrogen in the *γ* position (labelled in *m*), allows for the determination of the average molecular weight of PPG 2000. The value was found to be 1935 g/mol and agrees with the manufacturer's value of 2000 g/mol. The vinylogous urethane-modified PPG (mPPG 2000) was characterized after 24 hours of reaction and elimination of the by-product water. Compared with the PPG 2000 spectra, the modification leads to a downfield shift of the *α* hydrogens, labelled *k,* as in Figure S1, and an increase in peak *f*.

By comparing the integration of the hydrogens labelled *m* of PPG 2000 and mPPG 2000, the degree of functionalization of mPPG 2000 was calculated as 88.2% yield, which is 7% lower than the anticipated value of 95%. This could be due to a slight difference between the molecular weight of the manufacturer-provided PPG 2000 and the calculated value obtained from ^1^H-NMR.


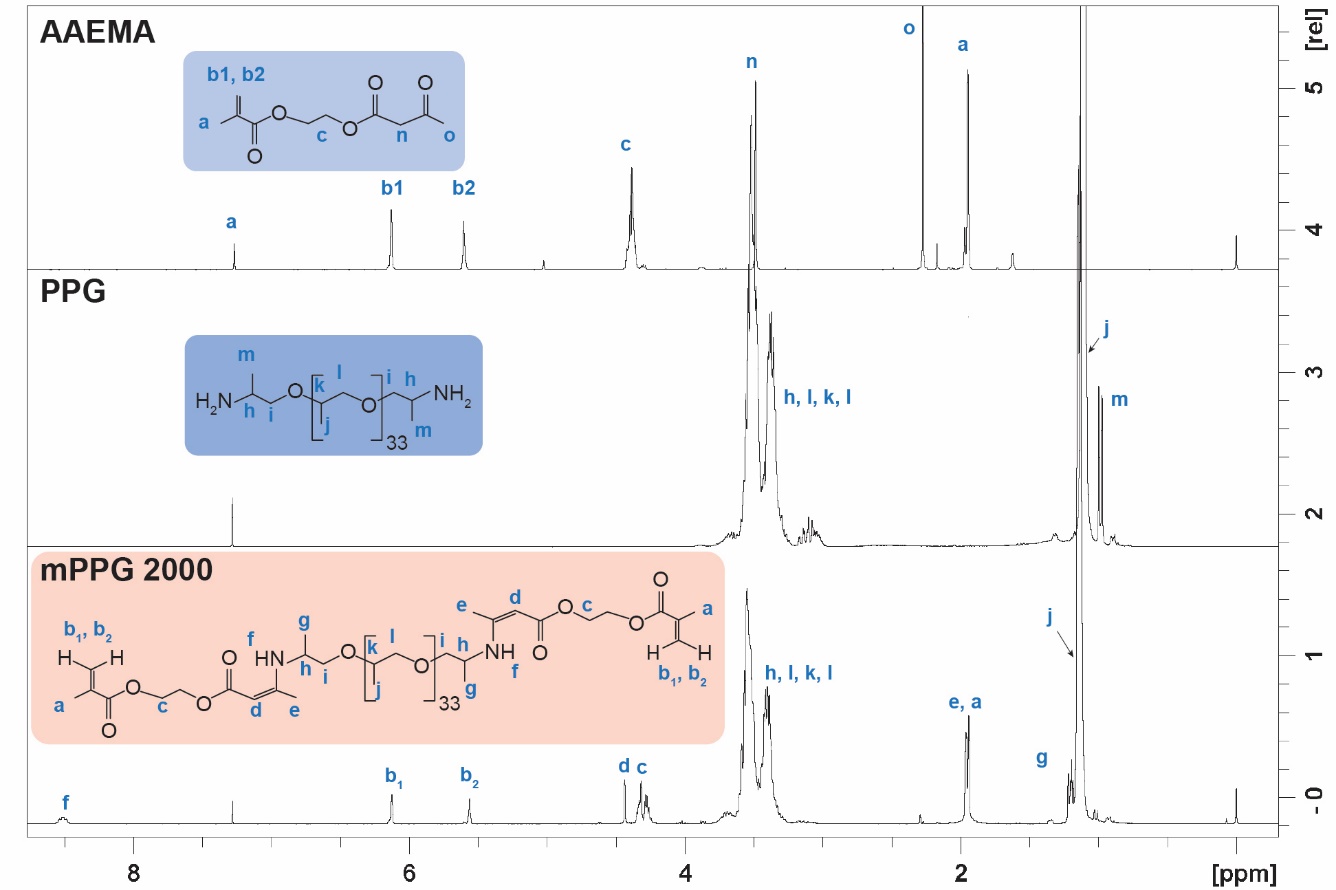


**Figure S1** ^1^H NMR (400MHz, CDCl3) spectra of AAEM, PPG 2000 and vinylogous urethane-modified PPG 2000 (mPPG 2000).

**Gel permeation chromatography**

The mPPG crosslinkers were analyzed via gel permeation chromatography (GPC) in chloroform solvent over a polystyrene column with poly (methyl methacrylate) calibration, with the lowest calibration weight of 1030 g/mol. All crosslinkers showed a peak molecular weight higher than that of the starting polymer. However, differences between modified / non-modified backbones were found to be in the range of 1100 - 3000 g/mol, which is much higher than the expected difference of 392 g/mol. As molecule separation in GPC is based on hydrodynamic volume, the functionalization of polymer backbones changes their chemical nature considerably, having a nonlinear influence on hydrodynamic volume and hence artificially distorting observed molecular weight values. To confirm this, a sequence of GPC measurements was performed on PPG 2000 and mPPG 2000, each sample being repeated three times. Such a method eliminates experimental variability such as sample preparation, temperature variation, column ageing, or contamination. **Figure S2** shows that traces for each compound were nearly identical.


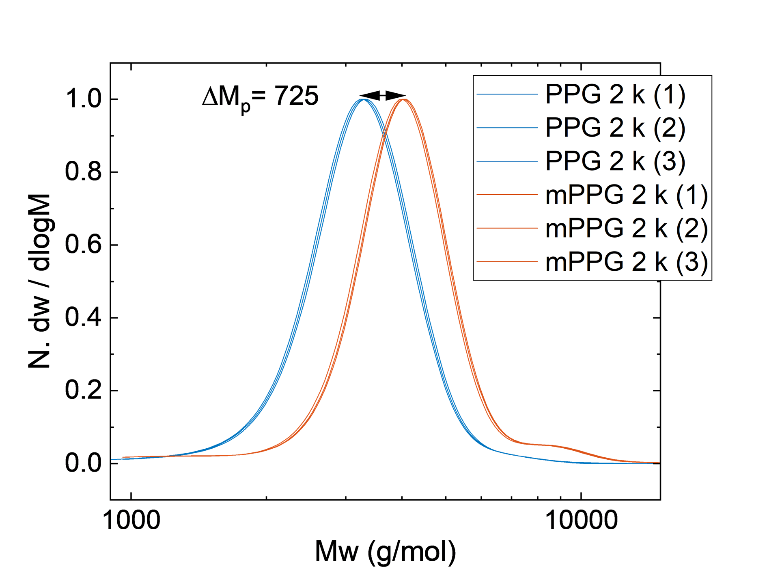


**Figure S2** Gel permeation chromatography (GPC) traces of PPG 2000 and mPPG 2000.

**3D-printing quality**

**Figure S3**A shows the worst surface quality on the tensile samples of 50 μm layer thickness, compared to the 30 μm sample. Figure S3B presents the bubble/voids on the 3D-printed self-healable gripper with a layer exposure time of 3.75 s, which will affect the function and mechanical performance of the gripper.


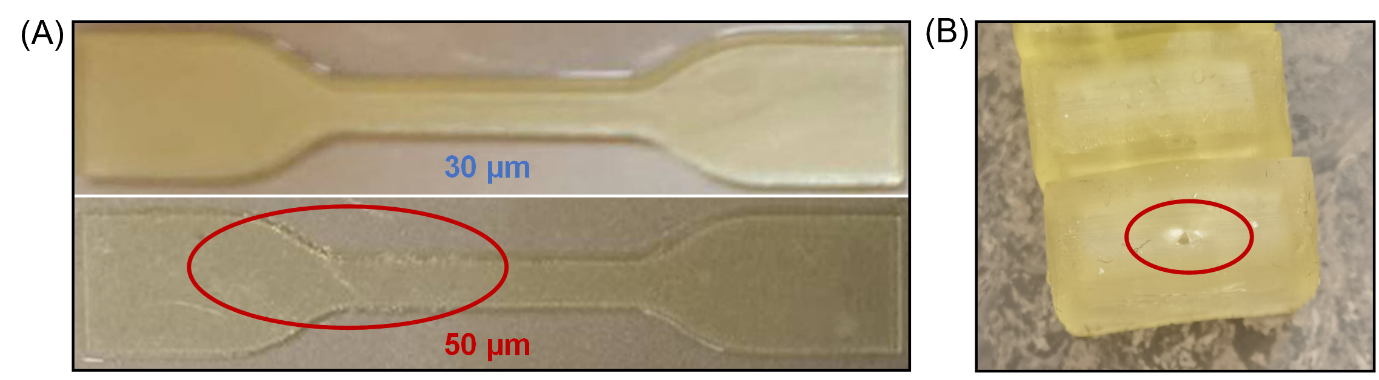


**Figure S3** Print quality using different 3D printing parameters. (A) Adhesion images of 3D-printed samples with layer thicknesses of 30 and 50 μm. (B) 3D-printed self-healable gripper with bubble/void with an exposure time of 5 s per layer.

**Stress relaxation and self-healing at 30 ℃**

**Figure S4** shows the stress relaxation test at 30 ℃, and the measured $\tau$ is 27705 s (7.7 h), 2.6 times longer than the theoretical value predicted by the Arrhenius equation. This deviation is attributed to the retarded recovery at room temperature. An SEM image of a broken then healed specimen after 24h at room temperature shows the disappearance of the crack and the recovery of the attached part.


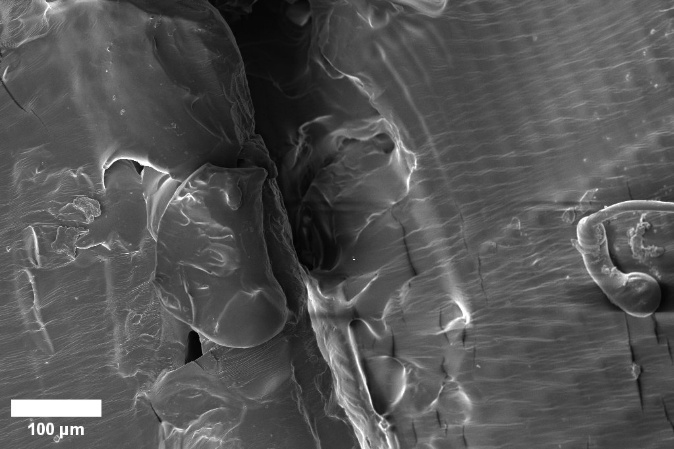


**Figure S4** Stress relaxation test of 3D printed specimen at 30 ℃ (left) and SEM image of the specimen after breakage and recovery for 24h at room temperature (right).

**Hysteresis testing of the material**

Hysteresis testing was undertaken, whereby five cycles were performed on four samples. Three of the samples were 3D-printed with polymer Formulations 1-3 (Table S2) and cured at 35 °C for 30 minutes, and another sample with Formulation 2 of mPPG 2000/EGMEA/IBOA (1.1 mol%/106/106) was cured at room temperature as a baseline (2 RT). The hysteresis test results are shown in **Figure S5**A-D. For each cycle, the clamped sample was stretched to an elongation of 100% and then retracted to the original position at a stretching speed of 50 mm/min. The hysteresis loss ratio can be calculated below:

$H_{i}=\frac{A_{l}}{A_{up}}$ (1)

Where *H_i_*, *A_up_,* and *A_l_* are the hysteresis loss ratio, the area under the uploading stress-strain curve, and the area of the loop curve, respectively, for the *i*_th_ cycle. The calculated hysteresis loss ratios in Figure S5D show that the formulation with a crosslink concentration of 1.1 (Formulation 2) has the highest hysteresis energy loss ratio over the five test cycles.


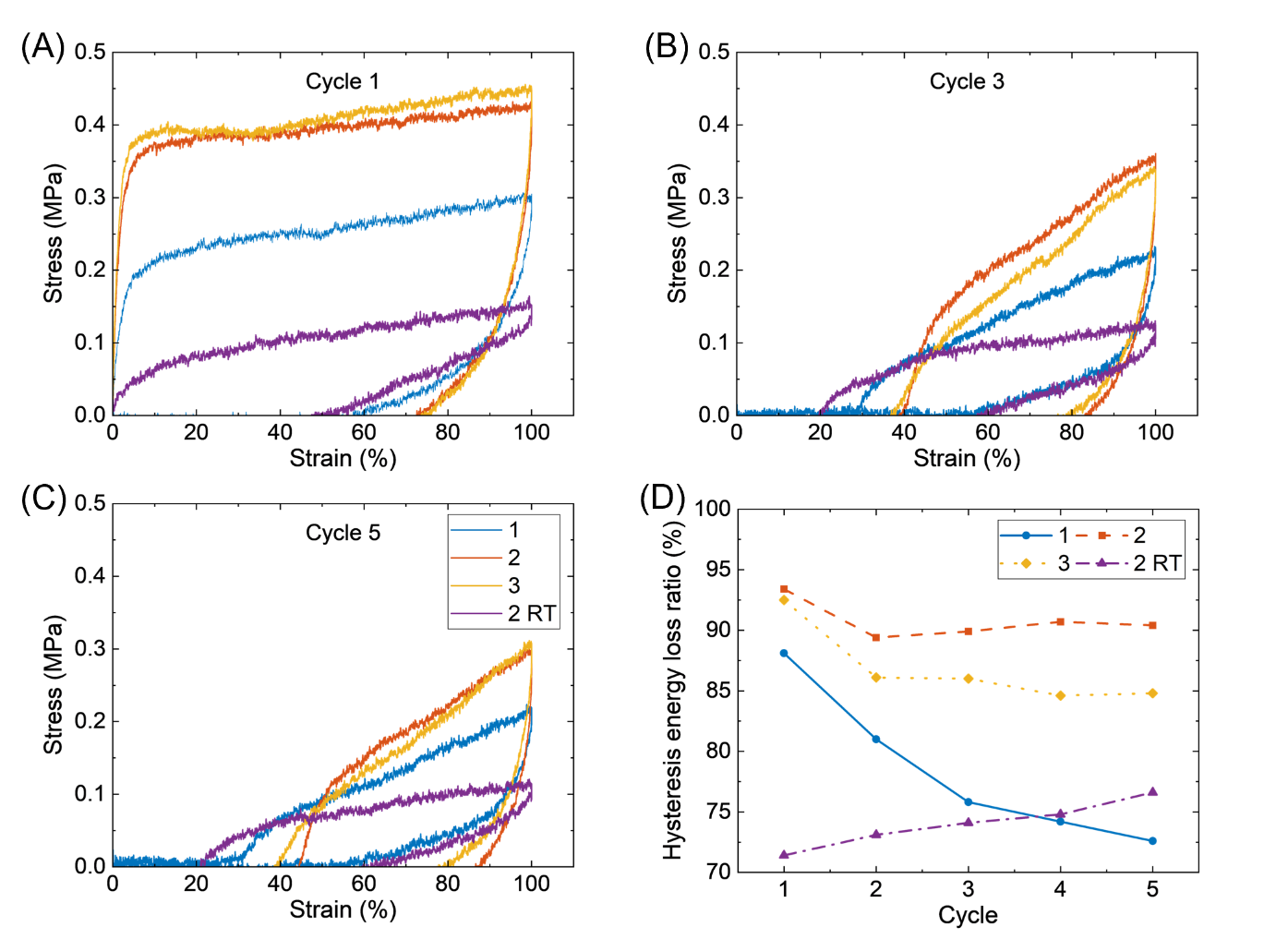


**Figure S5** Hysteresis tests of four selected samples. (A) Cycle 1. (B) Cycle 3. (C) Cycle 5. (D) Hysteresis energy loss ratio for four selected samples during five cycles.

**Gripper and crawler dimensions**

Detailed dimensions of the 3D-printed gripper and crawler used for simulation and 3D-printing are presented in **Figure S6**.


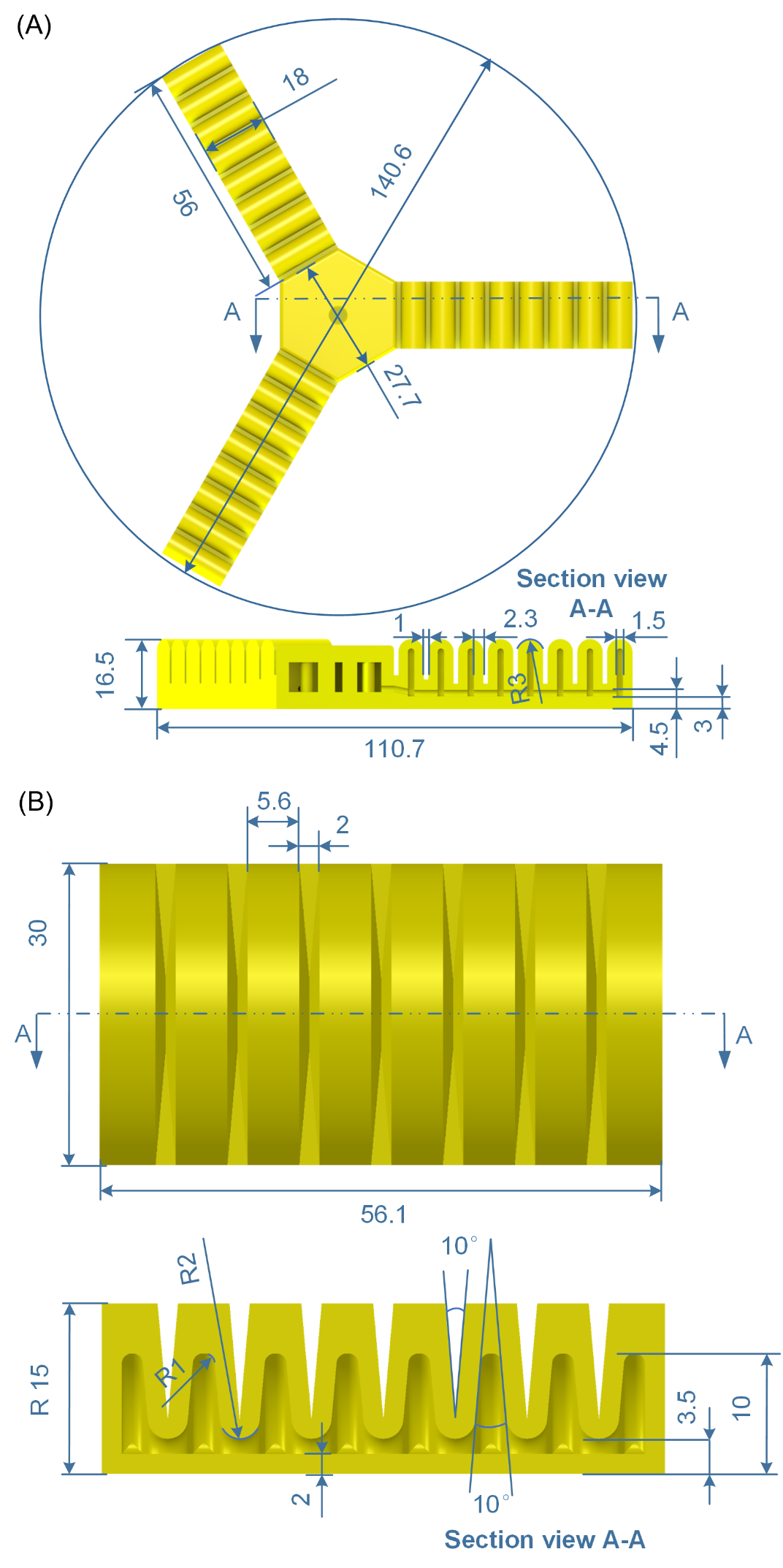


**Figure S6** Dimensions of the 3D-printed gripper and crawler. (A) Gripper design (unit: mm). (B) Crawler design (unit: mm).

**Finite element modelling (FEM) simulation in Ansys/Workbench**

The deformation of the 3D-printed self-healable soft gripper and crawler was modelled using a FEM model in Ansys/Workbench. The hyperelastic 3rd-order Yeoh model and the viscoelastic Prony Shear Relaxation model were used to model the material behaviour of the 3D-printed polymer. The material was considered incompressible, and gravity was taken into account by specifying the material density. The 3rd-order Yeoh model is given by:

$W=\sum_{i=1}^{N} C_{i}(\bar{I}_{1}-3)^{i}$ (2)

Where, *N* = 3, *C_i_* are the material constants identified by the experimental data, $\bar{I}_{1}=\lambda^{2}+\frac{2}{\lambda}$for uniaxial extension and *λ* is the principal stretch. The prony shear relaxation model is given by:

$G=G_{0}[\alpha_{\infty}+\sum_{i=1}^{n} \alpha_{i}\exp(-\frac{t}{\tau_{i}})]$ (3)

where, $G_{0}=G_{\infty}+\sum_{i=1}^{n} G_{i}$, $G_{0}$ and$G_{\infty}$ are the instantaneous moduli and the moduli at the slow limit. $\alpha_{\infty}=G_{\infty}/G_{0}$ and$\alpha_{i}=G_{i}/G_{0}$ are the relative moduli at the slow limit and at the relaxation time $\tau_{i}$.

Uniaxial tensile tests were conducted on a 3D-printed tensile sample to identify *C*_1_, *C*_2_, and *C*_3_, as shown in **Figure S7**A. The 3rd-order Yeoh model with *C*_1_ = 2.8835×10^5^, *C*_2_ = -6292.1, and *C*_3_ = 295.12 was fitted to the tensile test experiment data. The tensile tests were simulated in Ansys/Workbench using the fitted 3rd-order Yeoh model and the simulation result matched well with the experimental data. The stress relaxation tests were conducted and a 3-term Prony Shear Relaxation model with *α*_1_ = 0.02, *α*_2_ = 0.13, *α*_3_ = 0.25, *τ*_1_ = 1, *τ*_2_ = 10, *τ*_3_ = 100 was fitted to the test data, as shown in Figure S7B. However, some deviation was found between the simulated and experimental deformations of the gripper and crawler using the identified 3rd-order Yeoh model. This was found to be because the gripper and crawler were post-cured for the same amount of time (30 minutes) as the tensile test sample, but they were not cured to the same level due to their significantly different volume. To account for this effect, model parameters for the gripper and crawler were optimized to *C*_1_ = 16000, *C*_2_ = -700, and *C*_3_ = 30.645 and *C*_1_ = 65000, *C*_2_ = -1000, and *C*_3_ = 50, respectively. Similarly, the prony shear relaxation model parameters were optimized to *α*_1_ = 0.3, *α*_2_ = 0.15, *α*_3_ = 0.1 and *α*_1_ = 0.5, *α*_2_ = 0.15, *α*_3_ = 0.1 for the gripper and crawler, respectively, at *τ*_1_ = 1, *τ*_2_ = 10, *τ*_3_ = 100.


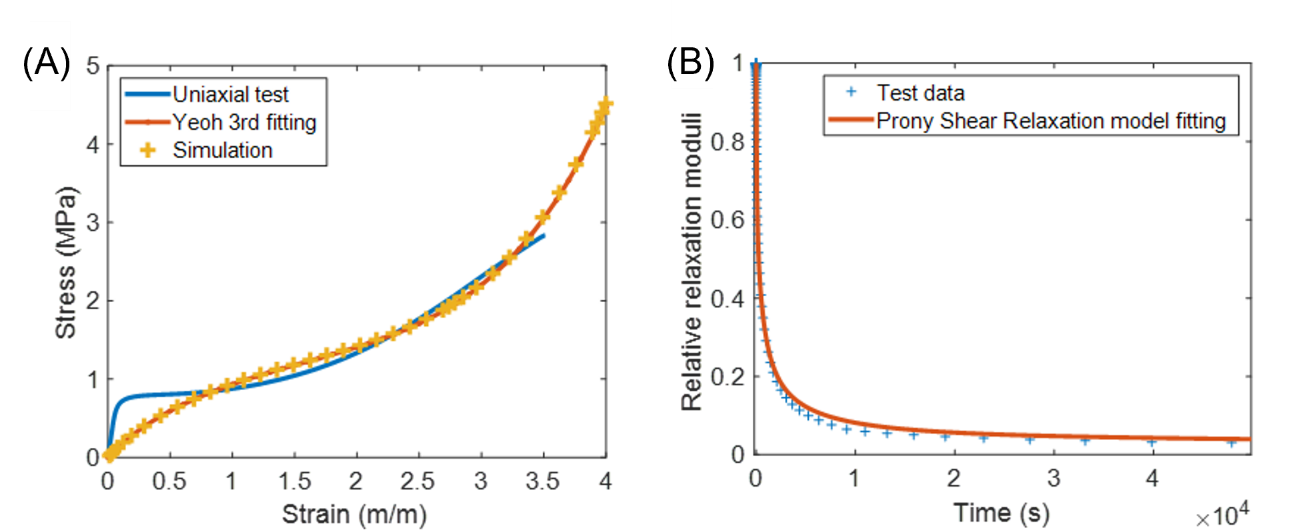


**Figure S7** Material modelling parameter identification. (A) 3rd-order Yeoh model parameter identification. (B) Prony Shear Relaxation model parameter identification.

**Experimental setups for supply pressure control**

The supply pressure to the soft robots was regulated by the pressure control system in **Figure S8**A. The pressure regulator regulates the maximum system pressure, with the compressed air from the central air compressing system. A proportional directional control valve (Festo MPYE) driven by a voltage signal controls the on/off and magnitude of the pressure to the soft robots. The flow transmitter (Festo Flow transmitter SFTE) and pressure transducer (Star Sensors, CYYZ11) measure the system air flow rate and output pressure. A closed-loop PID controller as shown in Figure S8B was designed in Matlab/Simulink and implemented on the Speedgoat Performance Real-time Target Machine. The output pressure was used as feedback to compare with the demand pressure, and the error between them is input to the PID controller. The saturation block limits the outputs of the voltage to the operating range of 4-10V of the proportional directional control valve, and the back calculation is chosen as the anti-windup method.


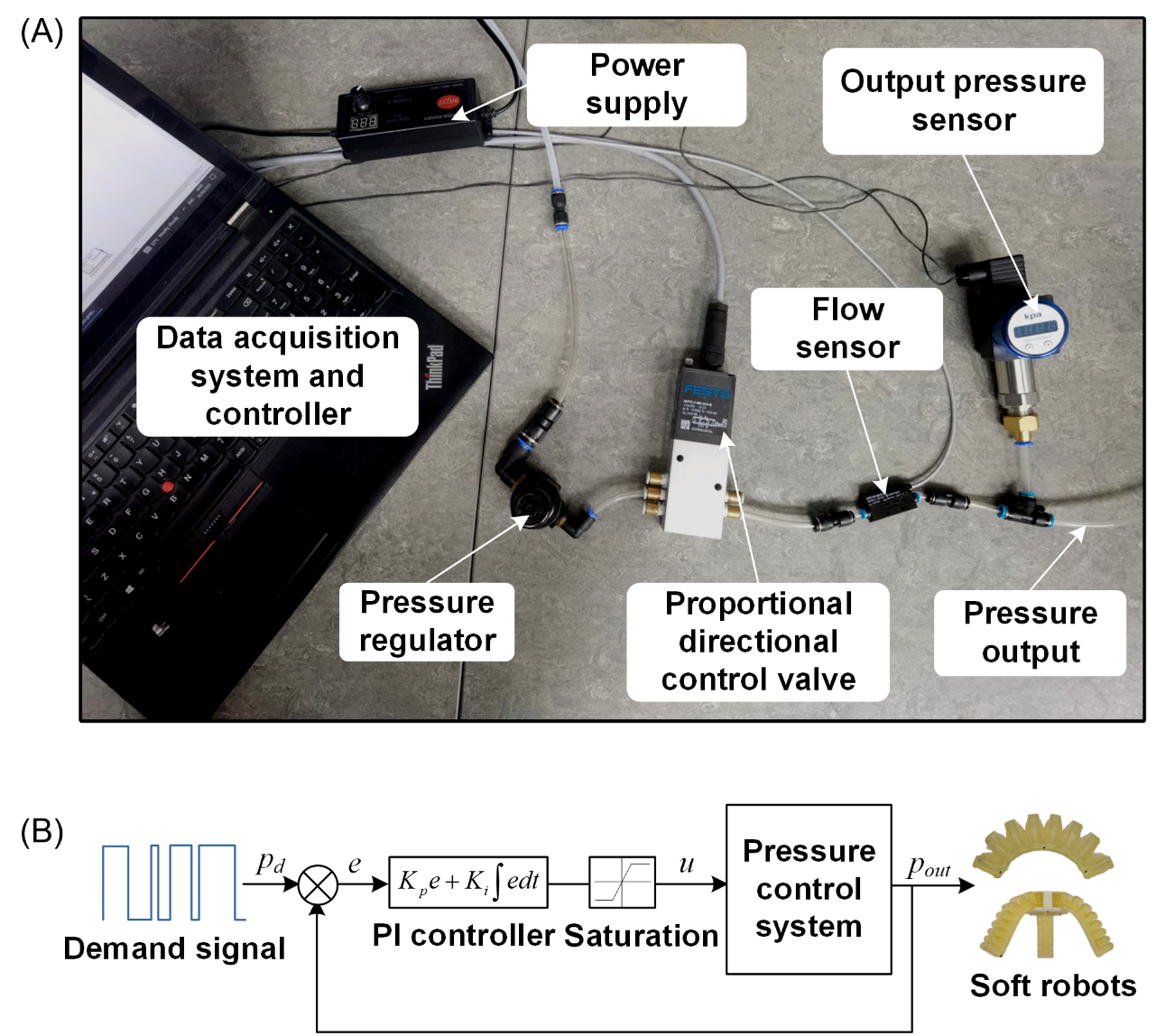


**Figure S8** Experimental setup for supply pressure control. (A) Pressure control system for mechanical characterization tests. (B) Closed-loop PID controller.

**Experimental setups for force and friction tests**

A customized experimental rig was built to characterize the static and dynamic tip force of the gripper, as shown in **Figure S9**A. A fingertip clamp was designed to hold the gripper finger, and a customized strain beam sensor measures the strain with a loading force and converts it to a voltage. The relationship between the loading force and voltage was calibrated with known weights and obtained by a cubic fitting, as shown in Figure S9B. A tensile force scale measures the friction force between the crawler with loaded weights and different surfaces to determine their friction coefficients, as shown in Figure S9C and D.


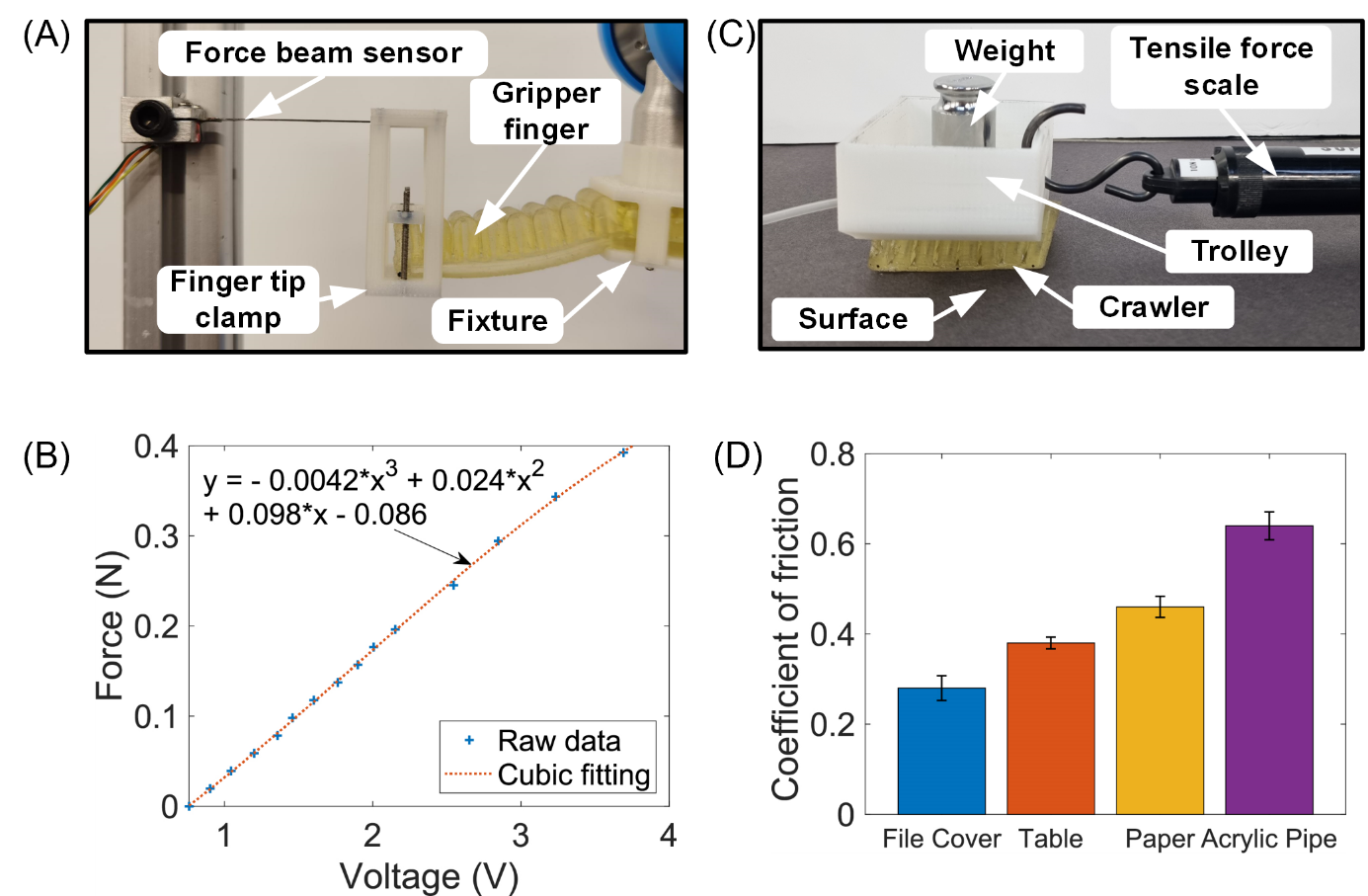


**Figure S9.** Experimental setups for force and friction tests. (A) Force tests. (B) Calibrated relationship between voltage and loading force of the force beam sensor. (C) Friction coefficient test rig. (D) Coefficients of different surfaces.

**Self-healing efficiency calculation**

We introduced a *self-healing efficiency* to quantitatively evaluate the self-healing capability of the 3D-printed soft robots after damage and healing as below:

$\eta=\frac{1}{n}\sum_{i=1}^{n} (1-\left|B_{i}-A_{i}\right|/A_{i})\times100$ (4)

where *A_i_* and *B_i_* are the test data before damage and after healing, and *n* is the number of data collected. The calculated self-healing efficiencies of the gripper and crawler are displayed in **Figure S10** and **Figure S11**. The self-healing efficiencies of the gripper static and dynamic displacement, and force tests range from 90.5% to 97% with an average of 94.5%. Despite the introduction of more severe levels of damage, the self-healing efficiencies of the crawler are only slightly lower than those of the gripper and achieve 77.3% to 94.1% with an average of 87.5%. The high self-healing efficiencies of the gripper and crawler evidence the excellent self-healing capability of the 3D-printed soft robot at room temperature after critical damage.


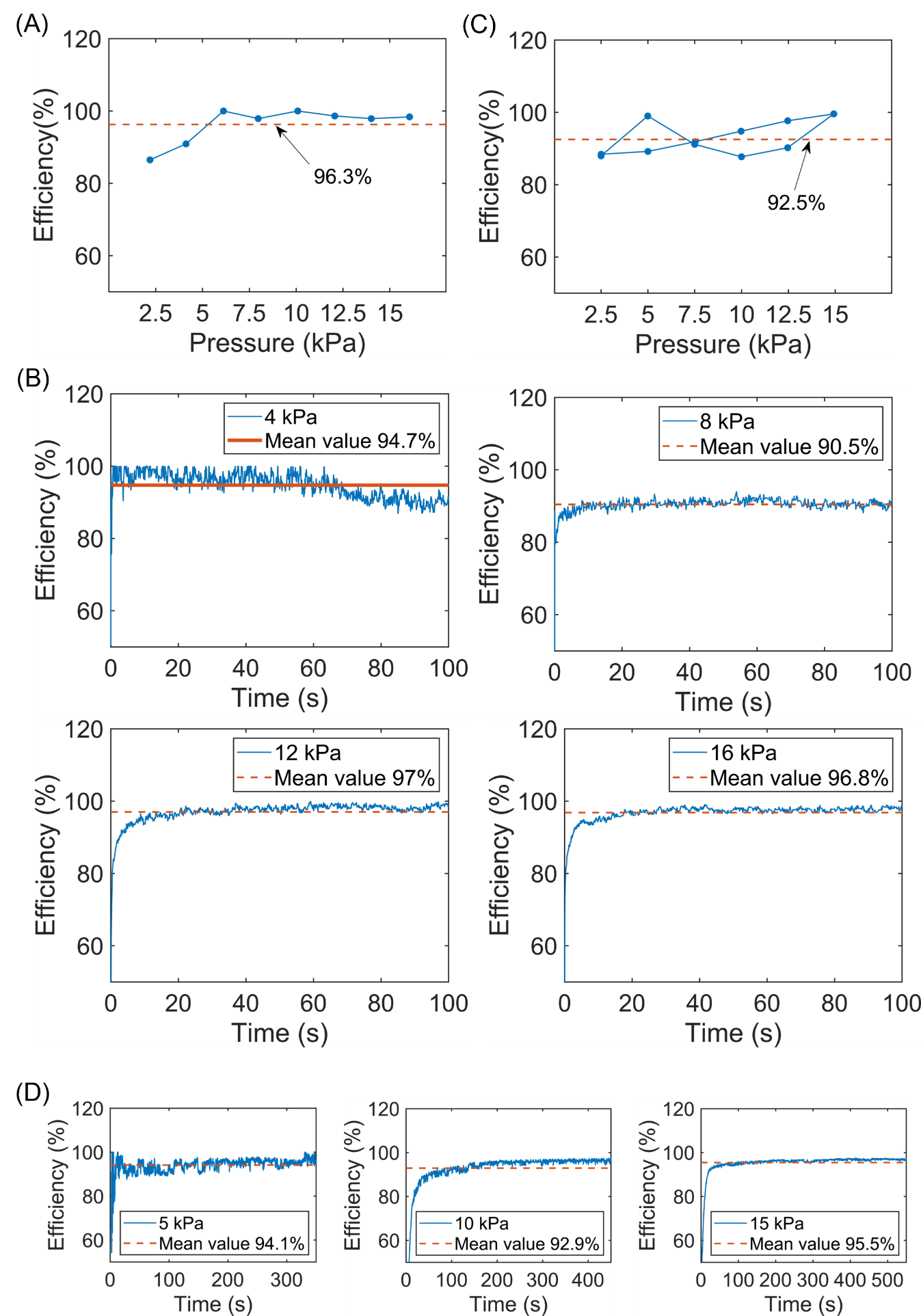


**Figure S10** Self-healing efficiencies of the 3D-printed self-healable gripper. (A) Static displacement. (B) Dynamic displacement at 4, 8, 12, 16 kPa. (C) Static force. (D) Dynamic force at 5, 10, 15 kPa.


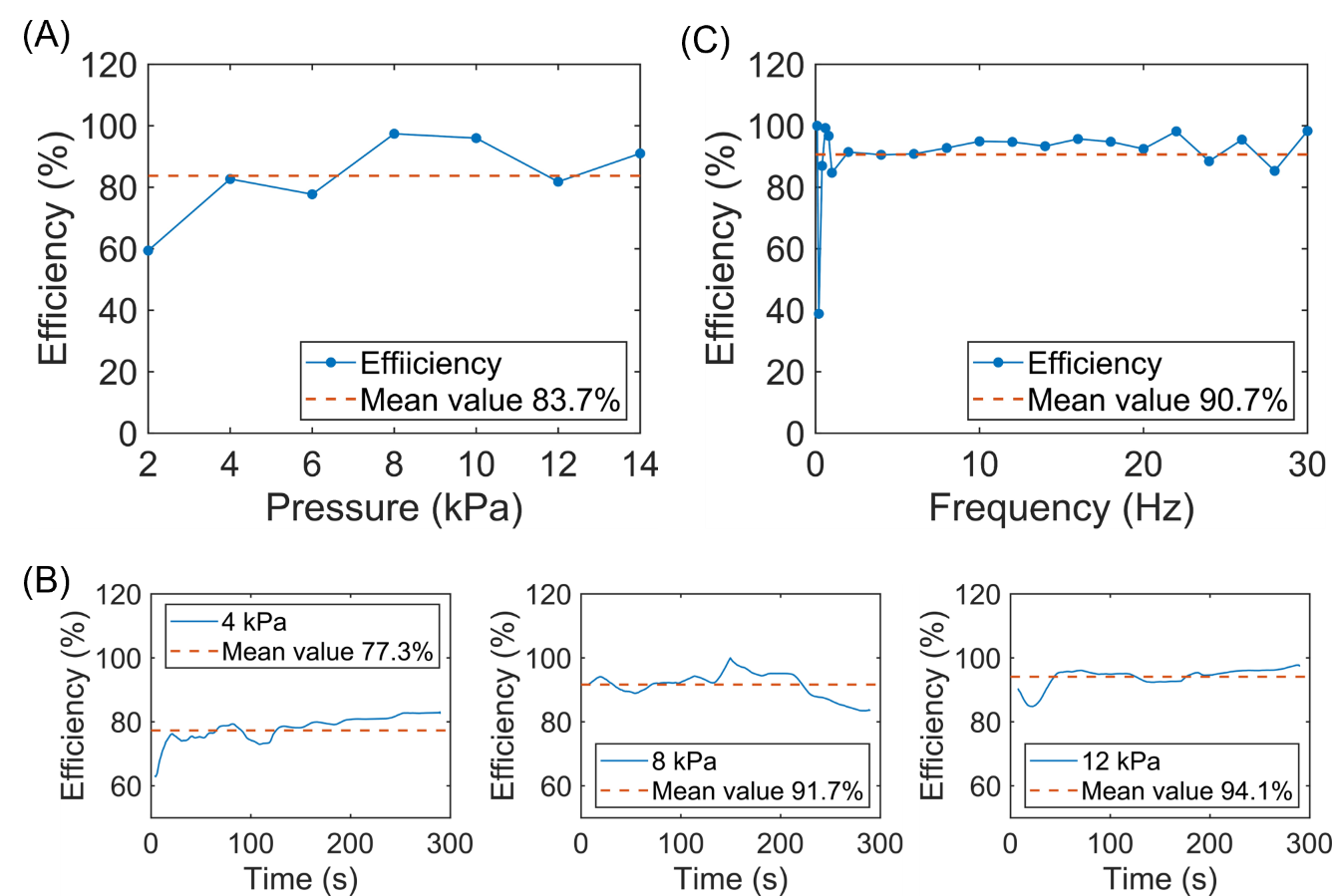


**Figure S11** Self-healing efficiencies of the 3D-printed self-healable crawler. (A) Static displacement. (B) Dynamic displacement at 4, 8, 12kPa. (C) Frequency test.

**Table S1.** Raw materials used in the formulated polymer

| Component | Description | Mass fraction (%) | Used in this paper |
| --- | --- | --- | --- |
| Monomer  (1 reactive site) | Provides the backbone of the polymer network | 85 - 98 | IBOA^a)^ (*T_g_* = 94 °C), EGMEA^b)^ (*T_g_* = -50 to -20 °C) ^[1-3]^ |
| Crosslinker  (≥2 reactive sites) | Provides functionality such as recyclability, stress relief, and elasticity | 1 - 12 | mPPG^c)^ 2000,  mPPG 4000,  mPPG 230 |
| Photoinitiator | Initiates free radical photopolymerization | 1 - 3 | BAPO^d)^ |

^a)^Isobornyl acrylate; ^b)^Ethyl glycol methyl ether acrylate; ^c)^Methacrylated diamine terminated poly (propylene glycol); ^d)^Phenylbis(2,4,6-trimethylbenzoyl) phosphine oxide.

**Table S2.** Polymer formulations for material optimization^*^

| Formulation  number | Crosslinker and  molecular weight (g/mol) | Crosslinker  mPPG^a)^  (molar ratio) | Monomer A  EGMEA^b)^  (molar ratio) | Monomer B: IBOA^c)^  (molar ratio) |
| --- | --- | --- | --- | --- |
| 1 | mPPG 2000 | 1 | 106 | 106 |
| 2 | mPPG 2000 | 1.1 | 106 | 106 |
| 3 | mPPG 2000 | 1.2 | 106 | 106 |
| 4 | mPPG 2000 | 1.5 | 106 | 106 |
| 5 | mPPG 2000 | 2 | 106 | 106 |
| 6 | mPPG 230 | 1.1 | 106 | 106 |
| 7 | mPPG 4000 | 1.1 | 106 | 106 |

*****BAPO photoinitiator concentration was kept constant as 2.5 wt% of the total weight of crosslinker and monomers for the above formulations; ^a)^Methacrylated diamine terminated poly (propylene glycol); ^b)^Methacrylated diamine terminated poly (propylene glycol); ^c)^Isobornyl acrylate.

**Table S3.** Formulation of vinylogous urethane-modified crosslinkers

| Reaction | PPG^a)^ oligomer | Mass of oligomer (g) | Mass of AAEGM^b)^ (g) |
| --- | --- | --- | --- |
| General synthesis  (90% functionalization) | 4000 | 68.29 | 6.95 |
|  | 2000 | 60.00 | 12.19 |
|  | 230 | 5.00 | 8.85 |

^a)^Poly (propylene glycol); ^b)^Acetoacetoxyethyl methacrylate.

**Table S4.** Mechanical properties, self-healing ability, and reprocessability of the elastomer in this work and others

| **Reference/**  **Parameters** | **This work** | **Yang et al.^[4]^ (2023)** | **Yang et al.^[5]^ (2024)** | **Zhu et al.^[6]^ (2025)** | **Yang et al.^[7]^ (2022)** |
| --- | --- | --- | --- | --- | --- |
| **Dynamic bond type** | Amine-enamine covalent bond | Imine  bond | Hydrogen bond | Boronic ester bond | Disulfide bonds |
| **Fabrication method** | Vat photopolymerization | Solution casting | Solution casting | Solution casting | Solution casting |
| **3D Printability** | Yes (layer thickness: 25–50 μm) | No | No | No | No |
| **Tensile strength** | 3.51 MPa | 2.51 MPa | 16.28 MPa | 3.17 MPa | 5.07 MPa |
| **Elongation at break** | 454% | 1158% | 660% | 1051% | 477% |
| **Healing method** | Room temperature (25 °C), 24 h | 60 °C, 6 h | 90 °C, 24 h | 80 °C, 24 h | 80 °C, 24 h |
| **Self-healing efficiency** | 94.5% (gripper function);  87.5% (crawler function) | 84.9% (sample tensile strength recovery) | 92% (sample tensile strength recovery) | 95% (sample tensile strength recovery) | 92.5% (sample tensile strength recovery) |
| **Reprocessing method** | Compression molding: 150 °C, 30 kN, 60 mins | Hot pressing: 80 °C, 20 MPa, 10 mins | Hot pressing: 140 °C, 20 MPa, 30 mins | Hot pressing: 140 °C, 20 MPa, 30 mins | Hot pressing: 170 °C, 20 MPa, 10 mins |
| **Tensile strength retention after reprocessing** | 70% after 1 cycle | 80.6% after 3 cycles | 91.4% after 2 cycles | 95.9% after 5 cycles | 60.9% after 2 cycles |

**Movie S1. 3D-printed self-healable soft gripper function demonstration**

**Movie S2. 3D-printed self-healable soft crawler moving in two-anchor mode**

**Movie S3. 3D-printed self-healable soft crawler moving in vibrational mode**

**Movie S4. 3D-printed self-healable soft crawler motion before cut and after healing**

**References**

1. J. Brandrup, E.H. Immergut, E.A. Grulke, Polymer Handbook. Wiley, New York.

2. N. Kurokawa, F. Endo, K. Bito, T. Maeda, A. Hotta, Antithrombogenic poly (2-methoxyethyl acrylate) elastomer via triblock copolymerization with poly (methyl methacrylate). Polymer 228, 123876 (2021).

3. M. Tanaka, T. Motomura, N. Ishii, K. Shimura, M. Onishi, A. Mochizuki, T. Hatakeyama, Cold crystallization of water in hydrated poly(2-methoxyethyl acrylate) (PMEA). Polymer International 49, 1709–1713 (2000).

4. Z. Yang, H. Li, X. Mou, Z. Chen, X. Lai, J. Ding, X. Zeng, Functional and environmental friendly polyimine elastomer based on the dynamic covalent network for a flexible strain sensor. Macromolecules 56, 9766-9777 (2023).

5. Z. Yang, S. Zhang, Z. Chen, X. Lai, H. Li, X. Zeng, Self-healing and degradable polycaprolactone-based polyurethane elastomer for flexible stretchable strain sensors. ACS Applied Polymer Materials 6, 905-914 (2024).

6. H. Zhu, X. Mou, Y. Guo, X. Lai, H. Li, X. Zeng, Self-healing and reprocessable chemically crosslinked polyurea elastomer based on boronic ester bonds for flexible strain sensor. Sustainable Materials and Technologies 45, e01636 (2025).

7. Z. Yang, H. Li, Y. Zhong, X. Lai, J. Ding, Z. Chen, X. Zeng, Functional epoxy elastomer integrating self-healing capability and degradability for a flexible stretchable strain sensor. ACS Applied Materials & Interfaces 14, 44878-44889 (2022).
